# Inverse Design of Pore Wall Chemistry To Control Solute Transport and Selectivity

**DOI:** 10.1021/acscentsci.2c01011

**Published:** 2022-11-30

**Authors:** Sally Jiao, Lynn E. Katz, M. Scott Shell

**Affiliations:** † Department of Chemical Engineering, University of California, Santa Barbara, California93106, United States; ‡ Department of Civil, Architectural and Environmental Engineering, University of Texas at Austin, Austin, Texas78712, United States

## Abstract

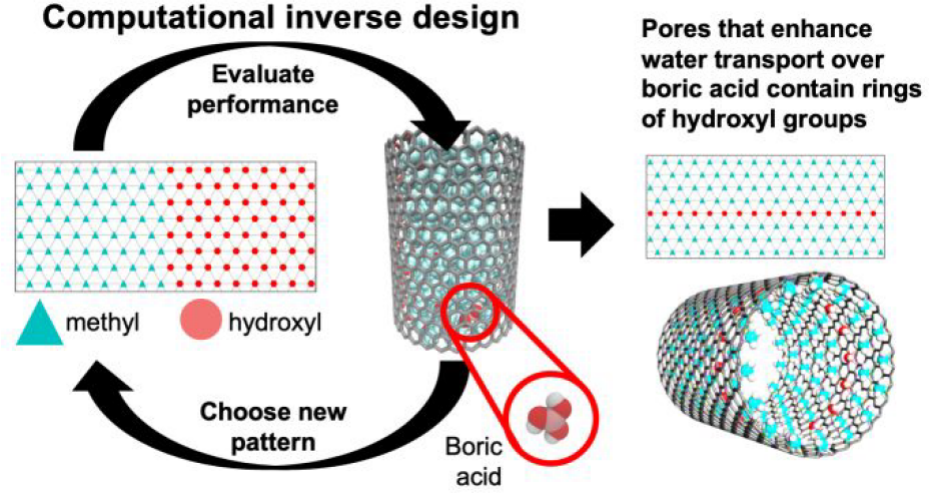

Next-generation membranes for purification and reuse of highly contaminated water require materials with precisely tuned functionality to address key challenges, including the removal of small, charge-neutral solutes. Bioinspired multifunctional membrane surfaces enhance transport properties, but the combinatorically large chemical space is difficult to navigate through trial and error. Here, we demonstrate a computational inverse design approach to efficiently identify promising materials and elucidate design rules. We develop a combined evolutionary optimization, machine learning, and molecular simulation workflow to spatially design chemical functional group patterning in a model nanopore that enhances transport of water relative to solutes. The genetic optimization discovers nonintuitive functionalization strategies that hinder the transport of solutes through the pore, simply by patterning hydrophobic methyl and hydrophilic hydroxyl functional groups. Examining these patterns, we demonstrate that they exploit an unexpected diffusive solute hopping mechanism. This inverse design procedure and the identification of novel molecular mechanisms for pore chemical heterogeneity to impact solute selectivity demonstrate new routes to the design of membrane materials with novel functionalities. More broadly, this work illustrates how chemical design is a powerful strategy to modulate water-mediated surface–solute interactions in complex, soft material systems that are relevant to diverse technologies.

## Abstract 2

Water scarcity is a grand challenge. Here, a computational inverse design approach discovers new, chemically patterned materials strategies that optimize the separation of contaminants from water.

## Introduction

The in silico inverse design of materials has long been a goal of computational modeling, promising accelerated materials discovery combined with reduced experimental cost. While system size and complexity have challenged earlier efforts, modern hardware and algorithms have given rise to many recent successes in the optimization of chemical functionalities. These efforts span a wide range of materials, from the design of small molecules,^1,2^ molecular complexes,^3^ and functionalized interfaces^4,5^ to self-assembling colloidal^6^ and polymeric systems.^7^ In some cases, existing structure–property relationships or learned empirical models simplify navigation of design space. Often, however, such models are inadequate or nonexistent, and molecular simulations are required to predict material properties, greatly increasing the computational expense of these design efforts. Soft material systems involving solution phases pose particularly difficult design problems that are due to often significant chemical heterogeneity, a concert of different interactions (excluded volume, electrostatic, van der Waals), and the emergence of complex driving forces involving entropies, such as hydrophobic interactions, that necessitate atomistic-level fidelity and long simulation time scales. Such systems often involve very large design spaces that are not easily enumerable or navigable as they lack obvious order parameters along which optimization can proceed, presenting additional challenges. There is therefore a pressing need for soft materials optimization approaches that make efficient use of generated simulation data, such as by incorporating on-the-fly learned models.^8^

A particularly important, complex class of materials that encompasses these challenges and is a major opportunity for computational soft materials design is water purification membranes. In this work, we demonstrate an inverse design approach to design the chemical functionality of a nanopore wall to optimize solute rejection in water separation membranes. We establish an optimization workflow coupling molecular simulations and machine-learned surrogate models and demonstrate its capabilities for complex soft material problems. We furthermore demonstrate how algorithm-generated materials can educe novel and nonintuitive design rules to guide synthetic efforts and advance the fundamental understanding of the relationship between surface chemistry and the transport properties of water and solutes.

Water scarcity will become an increasingly pressing issue in the coming decades.^9^ Energy security is intimately linked to the sustainability of current water resources,^10^ and thus, efficient water treatment processes are crucial for employing alternative lower quality water sources. The maturity of membrane technologies for low-energy, reliable treatment of seawater through reverse osmosis has led to their widespread adoption for desalination^11,12^ and utility for water reuse and other advanced water treatment systems.^13^ However, membranes are not yet capable of economically treating highly impaired waters, such as oilfield-produced water, currently an enormous wastewater stream on the order of 2 billion gallons per day.^14^ The development of membrane separation processes providing energy-efficient routes to converting such highly contaminated sources into “fit-for-purpose” water^15^ (e.g., for irrigation, streamflow augmentation, power) as well as recovering valuable resources from these waste streams remains a grand goal^16−19^ but is hindered by key challenges associated with the concentration and broad chemical variety of contaminants. For instance, the separation of small, charge-neutral solutes is particularly difficult for current membranes that leverage charge and size separation mechanisms.^16^ One exemplary solute is boric acid. While the World Health Organization guidelines recommend drinking water concentrations of less than 2.4 mg/L of boron,^20^ water reuse for agricultural irrigation of most crops requires even lower concentrations (<1 mg/L).^21,22^

Biology provides inspirational design considerations. Transmembrane proteins, such as aquaporin^23^ and potassium ion channels,^24^ achieve impressive permeability and selectivity through nanoscale confinement and precise arrangement of protein side-chain functional groups within the pores, shaping the chemical and topological landscape through which water and solutes transport. A number of efforts have made progress along this direction in synthetic systems.^25^ Nanochannels such as carbon nanotubes can exhibit unique^26,27^ and ultrafast transport of water,^28,29^ where the strength of the wall–water interaction modulates water transport.^30^ Functionalizing the pore also affects water adsorption^31^ and flux^32^ and modulates solute rejection,^33−37^ depending on the functionalization chemistry. Previous studies have also demonstrated that the location of the functional groups affects the water behavior^31,32^ and solute transport.^38^ However, a critical knowledge gap is understanding the potential impact of patterning pore surfaces with more than one type of functional group on engineering solute rejection and selectivity for the large class of neutral small-molecule solutes. The presence of more than one chemistry at a surface exponentially expands the design space. Indeed, previous computational work has shown that both the number and the arrangement of chemical functional groups at an extended surface can significantly tune the surface affinity of model solutes^5^ as well as the local diffusivity of water.^4^ Design rules and strategies to identify optimal, heterogeneous chemistries are largely unknown and would have implications beyond water filtration membranes to other chemically heterogeneous surfaces, such as porous adsorbents, catalytic surfaces, and antifouling interfaces, as well as heterogeneous macromolecules such as proteins and sequence-defined polymers.

In this work, as a case study, we focus on rejection of a neutral solute, boric acid, and demonstrate a novel workflow to precisely tune membrane pore wall spatial chemistry to enhance solute rejection. While Edisonian approaches in the membrane community have identified important heuristic membrane material characteristics affecting, e.g., fouling^39,40^ and solute uptake,^41−44^ such approaches only skim the large design space associated with multifunctional chemistries. Instead, here, we describe a computational inverse design workflow that optimizes the relative transport of water over boric acid, merely by patterning nonpolar methyl and polar hydroxyl groups tethered to a model pore. This proof of concept demonstrates the potential of computational materials discovery in uncovering the existence of nonintuitive, heterogeneous pore wall chemistries that enhance solute rejection. In particular, the optimization workflow suggests that pores with rings of hydroxyl groups pose selective barriers to boric acid transport over water, suggesting a new class of materials to explore for water separations. On the basis of these computationally designed pore surfaces, we identify a mechanism behind the enhancement in boric acid rejection captured by a simple, predictive analytical model. We then demonstrate that the optimal pore design gives enhanced selectivity for a range of other solutes that vary with respect to polarity, hydrogen-bonding capability, and surface affinity, suggesting the generality of the proposed mechanism.

## Results and Discussion

### Genetic Algorithm Optimization

**Figure 1:**
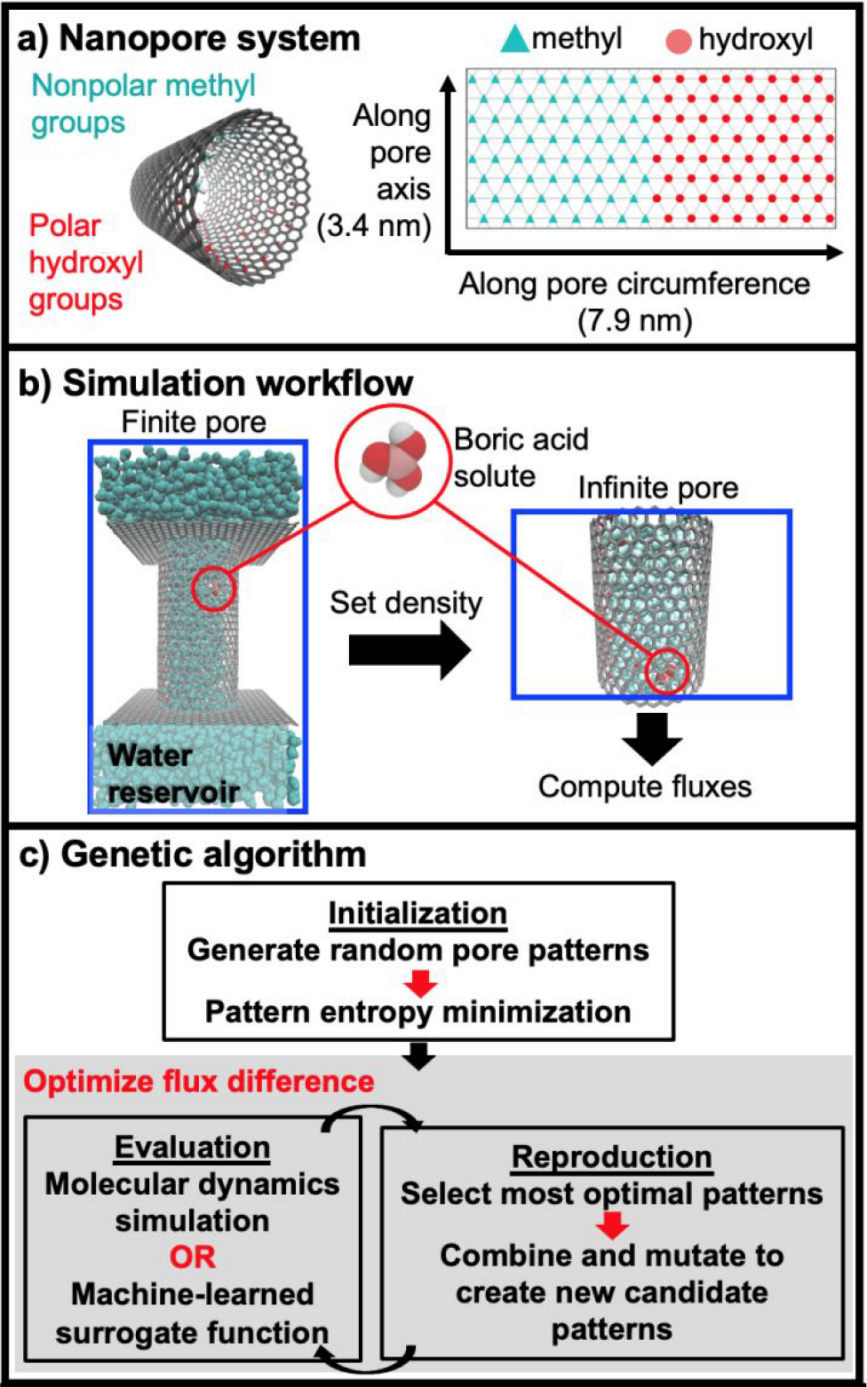
(a) Model nanopore system allows for precise patterning of nonpolar methyl groups and polar hydroxyl groups along the CNT backbone. For ease of visualization, we present the flattened pore patterns with blue triangles representing methyl groups and red circles representing hydroxyl groups. (b) We first simulate the finite pore to compute the density of water, which we then set in the infinite pore to compute water and boric acid transport properties. (c) A genetic algorithm incorporating MD simulations and a learned surrogate function allows for automated identification of pore wall chemical patterns optimizing pore transport properties.

Our basic question is: to what extent can surface chemical patterning be leveraged to optimize the relative transport of water versus neutral solutes in a pore? Here, we use boric acid as a model solute, which is difficult to separate due to its small size and charge neutrality at neutral pH, and we consider nanopores of varied hydrophobic/hydrophilic content, tuned by patterning the interior of a carbon nanotube (CNT) with hydroxyl and methyl functional groups (Figure 1a). To compute transport properties of water and solutes inside the pore, we first simulate a “finite” pore attached to a water reservoir to equilibrate the density of water inside the pore and then simulate an “infinite” pore with the same density (Figure 1b) from which we compute water and boric acid transport metrics, such as equilibrium diffusivity and nonequilibrium, pressure-driven flux (details in Methods). To assess the potential of chemical patterning to enhance pore transport of water relative to boric acid, we use a bioinspired evolutionary or genetic algorithm (Figure 1c) that identifies arrangements of methyl and hydroxyl functional groups on the pore wall that optimize the difference between the computed water and boric acid fluxes. To accelerate the optimization, we employ a machine-learned surrogate objective function trained on prior explicitly simulated patterns to predict the flux difference. The surrogate function is periodically retrained with additional batches of explicit molecular dynamics (MD) calculations. The surrogate model allows exploration of orders of magnitude more generations than is possible with MD alone due to computational expense. Because we use a linear model in the feature space, the surrogate model also serves to average out statistical noise from the MD-computed flux, giving the genetic algorithm robustness to uncertainty (Figure S14).

**Figure 2:**
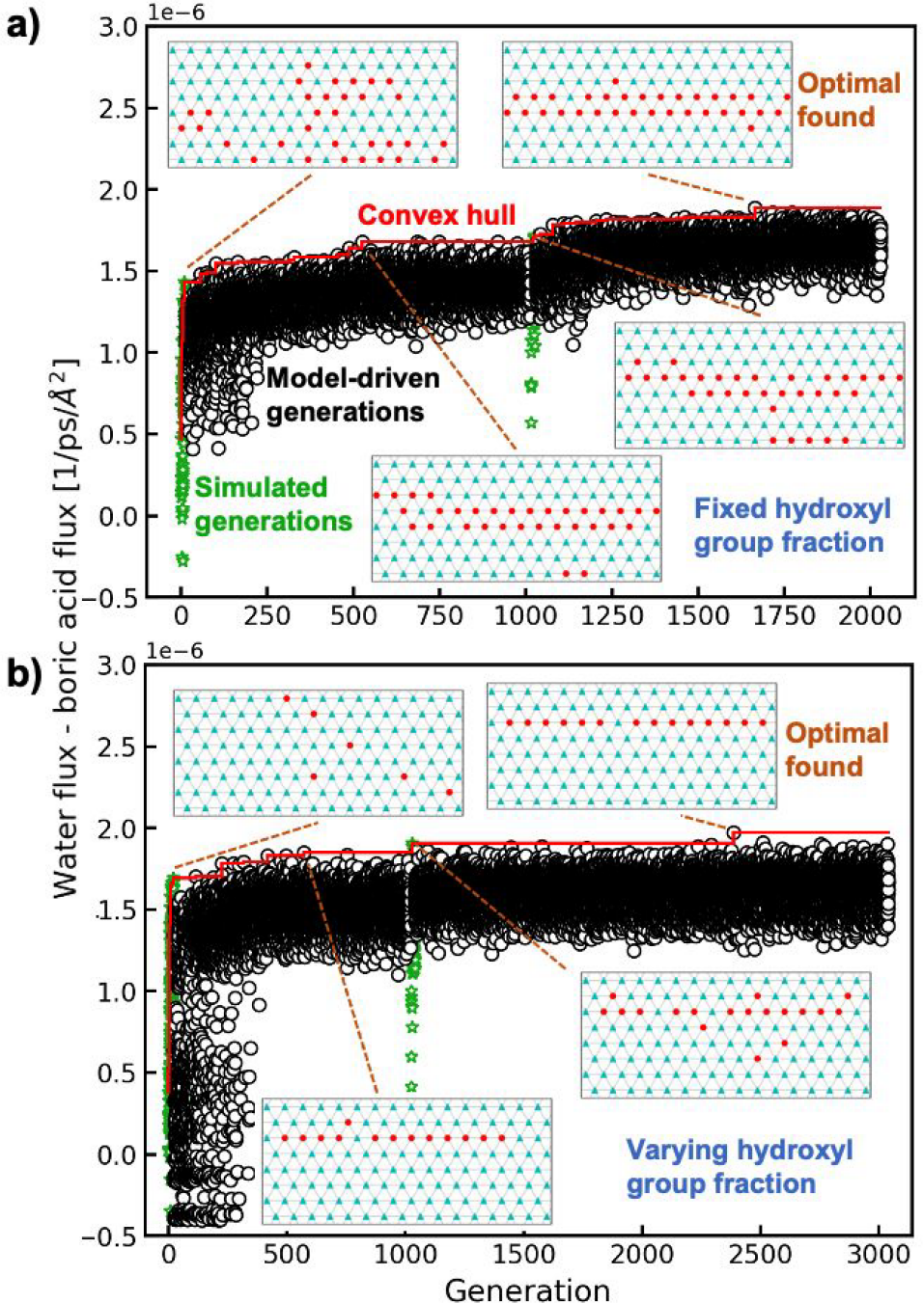
Genetic algorithm trajectories maximizing the difference between water flux and boric acid flux for (a) fixed hydroxyl group fraction and (b) varying hydroxyl group fraction show optimization to surfaces with rows of hydroxyl groups. Each marker corresponds to a pattern discovered by the genetic algorithm. Simulated generations are shown as green stars and surrogate model-driven generations as black circles. Inset patterns show the optimal patterns found after (1) the first set of simulated generations, (2) the first set of model-driven generations, (3) the second set of simulated generations, and (4) the second set of model-driven generations. The red line shows the maximum value of the objective function at each point in the optimization.

This optimization approach detects novel functionalization strategies: Figure 2 shows the trajectory of the flux difference versus the number of generations for genetic algorithm runs fixing the hydroxyl group fraction at 0.25 (32 hydroxyl groups) (Figure 2a) and allowing the hydroxyl fraction to vary (Figure 2b). Both trajectories span a large range of flux differences and thus demonstrate that chemical patterning can be tuned to enhance solute rejection. The optimal pattern found at a fixed hydroxyl fraction contains two nearly perfect rings of hydroxyl groups (shown in the 2D schematic as rows), while the variable hydroxyl case with a much larger search space identifies an optimal pattern that contains a single ring of hydroxyl groups. The optima suggest that rings of hydroxyl groups inside the nanopore particularly inhibit boric acid transport with respect to water transport, a nonobvious result. Patterns that include these features are not identified in the first set of simulated generations, showing that the surrogate function is key to exploring a sufficiently large number of generations to identify these optimal motifs.

The surrogate model is key to enabling high-throughput optimization. We find that it only needs to maintain accuracy in predicting the flux difference of patterns similar to those currently explored by the genetic algorithm (“local” in pattern space) to enhance performance, with periodic retraining through new MD simulations ensuring its ongoing relevance. Figure S15 shows that the linear model is indeed able to fit the “training” data (a single set of explicit MD simulations) well, suggesting good local accuracy. The model also predicts the flux difference of patterns outside of the training set qualitatively well, giving accurate directional trends, which is the key requirement to advance the genetic algorithm. The quantitative accuracy is also reasonable given the statistical uncertainty associated with the MD simulations (Figure S15). Beyond the linear model, we find that neural-network-based surrogates do not perform significantly better (Figure S16) and furthermore require more extensive hyperparameter selection while sacrificing interpretability. We instead take advantage of the interpretability of simpler models (specifically, LASSO and random forest regression) to compute feature importance metrics demonstrating that spatial correlations of hydroxyl groups along the pore axis and circumference, and especially rows of hydroxyl groups, are the most important features for the regression (Table S4), reinforcing the idea that these motifs are significant.

### Behavior of Rationally Designed Patterns Suggests Mechanism for Boric Acid Rejection

**Figure 3:**
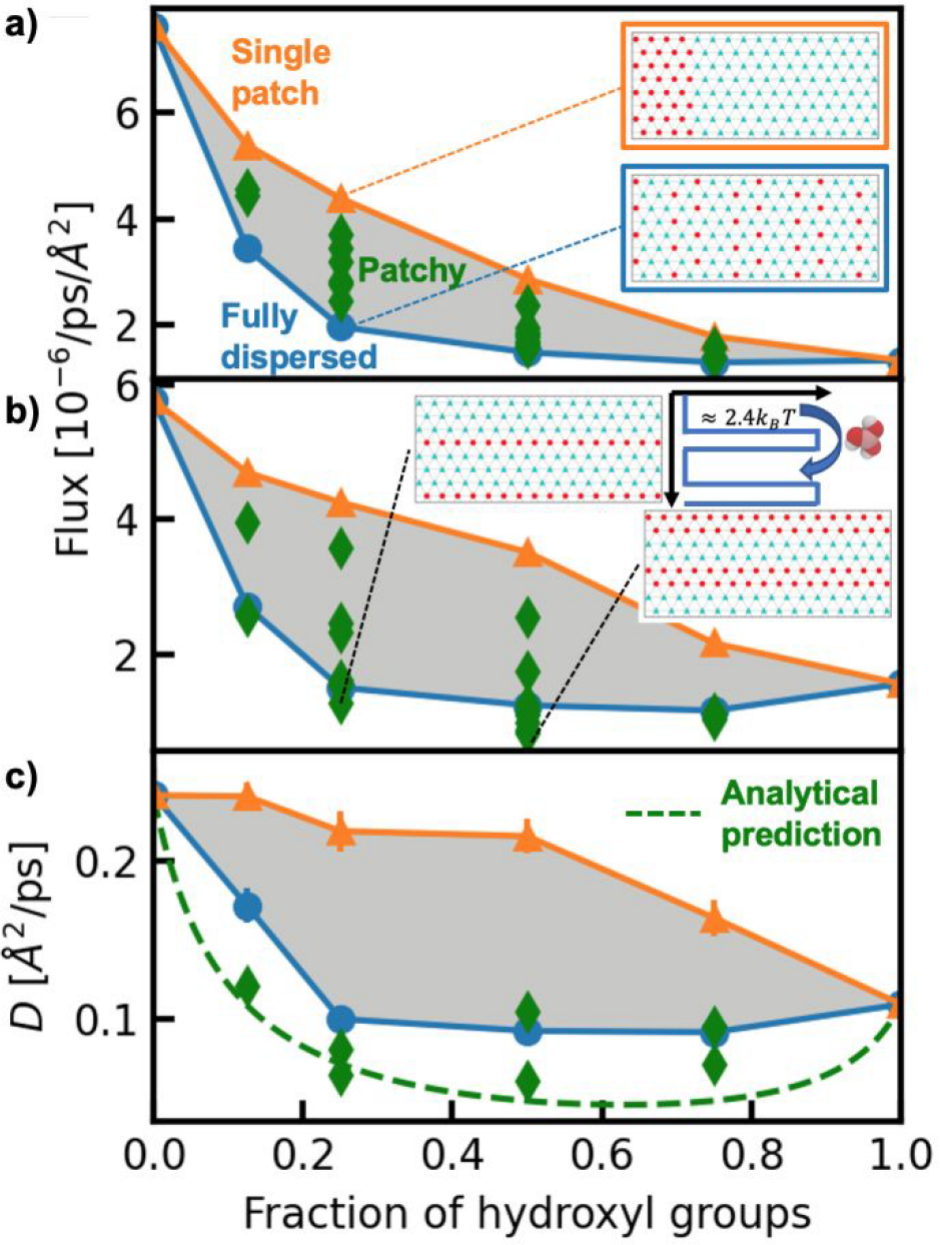
Pattern of methyl and hydroxyl groups has a significant effect on the flux of (a) water and (b) boric acid in nonequilibrium simulations and on (c) the diffusivity of boric acid in equilibrium simulations. For each panel, orange triangles represent patterns with a “single patch” of hydroxyl groups, spanning the pore axis, while blue circles represent “fully dispersed” patterns (examples of both are in the inset of a). Green diamonds represent other patterns where the hydroxyl groups form smaller patches (“patchy”). Two examples of such patterns are shown in the inset of b. These patterns also show lower boric acid flux compared to the fully dispersed patterns. The inset also includes a schematic of the periodically varying free energy landscape used in the analytical model to predict the reduction in boric acid diffusivity in the ringed pores. In c, green diamonds are only shown for patterns with rows of hydroxyl groups. The dashed line shows the analytical prediction described in the text based on a model of the periodic free energy landscape depicted in the inset of b.

Inspired by the genetic algorithm results, a rational exploration of the effect of pore patterning on water and solute transport reveals a rich design landscape. Figure 3 visualizes this design space, by showing the water flux (Figure 3a) and boric acid flux (Figure 3b) as a function of hydroxyl fraction in regularly patterned pores (Table S5), chosen as idealized versions of the motifs identified from the genetic algorithm-discovered patterns (Figure S17). Figure 3a clearly illustrates that the pore wall chemistry significantly modulates water transport: increasing the number of hydroxyl groups generally reduces water flux, with the fully methylated and fully hydroxylated pores capturing the extreme cases with an approximately 4-fold difference in flux. However, even at a fixed fraction of hydroxyl groups, the specific pattern of the groups also significantly affects transport: for instance, patterns with a hydroxyl group fraction of 0.25 span a 2-fold change in flux difference. In contrast, previous work that “patterned” a CNT with chemically unspecific LJ sites of varying wall–water interaction strengths revealed that functional group arrangement did not affect water flux.^45^ The present study thus suggests that functionalization with chemically specific moieties involving, e.g., hydrogen-bonding interactions is required to observe pattern-dependent trends, consistent with previous work showing the dependence of water diffusivity on surface patterning.^4^

In particular, the patterns with the hydroxyl groups in a single patch spanning the pore axis (orange triangles) lead to maximal water flux out of all patterns examined, while those that disperse the hydroxyl groups (blue circles) produce minimal flux; intermediate behaviors are seen from patterns with hydroxyl groups arranged in multiple, smaller patches (green diamonds). The diffusivity of water in these pores from equilibrium simulations recapitulates the same nonequilibrium flux trends (Figure S18). These behaviors are consistent with earlier observations at extended self-assembled monolayer surfaces in which both increasing the number of hydroxyl groups and dispersing them more evenly across the surface lead to a reduction in surface water diffusivity.^4^ More broadly, the pattern-dependent trends follow similar findings for water near surfaces where increased dispersion of polar sites leads to reduced surface hydrophobicity, which manifests as reduced water density fluctuations,^46,47^ reduced tendency to dewet,^48−50^ and reduced solute affinity.^5^

The design space for boric acid in Figure 3b shows that the pore wall chemistry generally affects its transport similarly to water; however, the patterns that minimize boric acid transport for a given hydroxyl fraction are not fully dispersed but instead contain rows of hydroxyl groups (Figure 3b, inset). These motifs use rings of hydroxyl groups to reduce boric acid flux *relative* to water (Figure S19), rationalizing the optimal surfaces located by the genetic algorithm. For the ringed pores (Figure 3b, inset), boric acid flux is less than half that of water, a reduction that is sufficient to reduce boric acid concentration in seawater (4.5 mg/L)^21^ to the target concentration for drinking water (2.4 mg/L).^20^ We note, though, that treating groundwater, which can contain higher concentrations of boric acid, or achieving a lower target concentration for irrigation water would require recycling.

To understand the origin of this unique behavior, we hypothesize that two mechanisms are important: (1) boric acid strongly partitions to the hydrophobic, methylated regions of the pore wall, and (2) it is then forced to “hop” over the hydroxyl rings with an energy barrier determined by rearrangements to the hydrogen-bonding network. Boric acid, while highly polar, shows affinity for methylated wall sections of the pore due to its small size; earlier simulation results demonstrated that water drives boric acid to methylated surfaces as a way to minimize the penalty associated with water restructuring around the solute in bulk solution distant from the interface.^5^ To assess this hypothesis, we compared measurements of the diffusivity from equilibrium simulations to predictions of an analytical model from ref (51) that describes the 1D diffusive transport of a particle (here, boric acid) in a periodic energy landscape (Figure 3b, inset). We estimate the height of the free energy barrier from moving between methylated sections (across hydroxylated sections) to be 2.4*k*_B_*T*, based on the partition coefficients of boric acid into fully methylated versus fully hydroxylated pores. The periodicity of the landscape is then set by the geometry of the pattern, i.e., the number of methylated and hydroxylated rows (details in section S5D, Supporting Information). We combine this prediction with a simple exponential decay that describes the background reduction in diffusivity due to the surface hydroxyl group fraction (section S5D, Supporting Information). The resulting model then predicts the boric acid diffusivity (Figure 3c, dashed line) in agreement with equilibrium simulations for pores with various arrangements of rows of hydroxyl groups. This mechanism is consistent with earlier work on the reduction in water dynamics in rough versus smooth pores, concluding that the primary mechanism is the creation of a rough energy landscape.^52^

At higher fractions of hydroxyl groups (0.50 and 0.75), not all patterns with rows of hydroxyl groups agree with the analytical prediction. We hypothesize that at least two consecutive rows of methyl groups are required to produce an effective hydrophobic surface environment, as previous work has shown that polar groups affect the local water behavior near adjacent methylated regions^48^ such that isolated methyl groups have little effect on the local water behavior.^47^ Consistent with this hypothesis, the patterns showing the largest deviations from the analytical model each contain isolated single rows of methyl groups. At lower hydroxyl group fractions, the agreement between the analytical model and the MD diffusivities is consistent with the proposed hopping mechanism, reinforcing this potential route to inhibiting solute transport while maintaining water transport. These observations underscore the relevance of nonadditive, context-dependent interactions to the design of hydrated systems. Here, the arrangement of hydroxyl groups affects solute transport, independent of the average density of the hydroxyl groups and even beyond basic characterization of the pattern (i.e., degree of clustering), thus demonstrating behaviors that are highly nonlinear on a nanoscopic scale.

Transport through the pore is not the sole factor affecting membrane performance. Water and solutes must first partition into it: both the number and the arrangement of hydroxyl groups affects water and boric acid partition coefficients (Figure S21). While patterns with more hydroxyl groups or dispersed hydroxyl groups lead to enhanced water partitioning and reduced boric acid partitioning and flux, they also produce reduced water flux, suggesting a trade-off between permeability and selectivity, a commonly observed relationship for other membrane materials that captures how improvements to solute rejection tend to be at the expense of reducing throughput.^41^ This trade-off is a key challenge for membrane design efforts. To assess this behavior, we compute the permeability from water’s partition coefficient (tendency to go into the pore) multiplied by its in-pore flux (transport once inside the pore). We compute the selectivity from the ratio of the permeability of water to that of boric acid. The calculated permeability and selectivity values generally reinforce the trade-off: for instance, water permeability increases from the fully hydroxylated pore (0.703_4_ mol-nm/cm^2^-s-MPa) to the pore with an equal number of hydroxyl and methyl groups arranged in single, axial patches (Figure 1a) (1.300_4_ mol-nm/cm^2^-s-MPa) and again to the fully methylated pore (2.866_8_ mol-nm/cm^2^-s-MPa). However, selectivity for water over boric acid decreases along that same series (57_1_ × 10^4^, 9.3_2_ × 10^4^, and 5.8_3_ × 10^4^). Beyond these specific patterns, randomly generated pores also demonstrate this trade-off (Figure S22). However, the optimal genetic optimization patterns display improved permeability–selectivity behavior compared to randomly patterned pores (Figure S22), suggesting that optimization of the flux difference may produce pattern motifs that address the permeability–selectivity trade-off.

**Figure 4:**
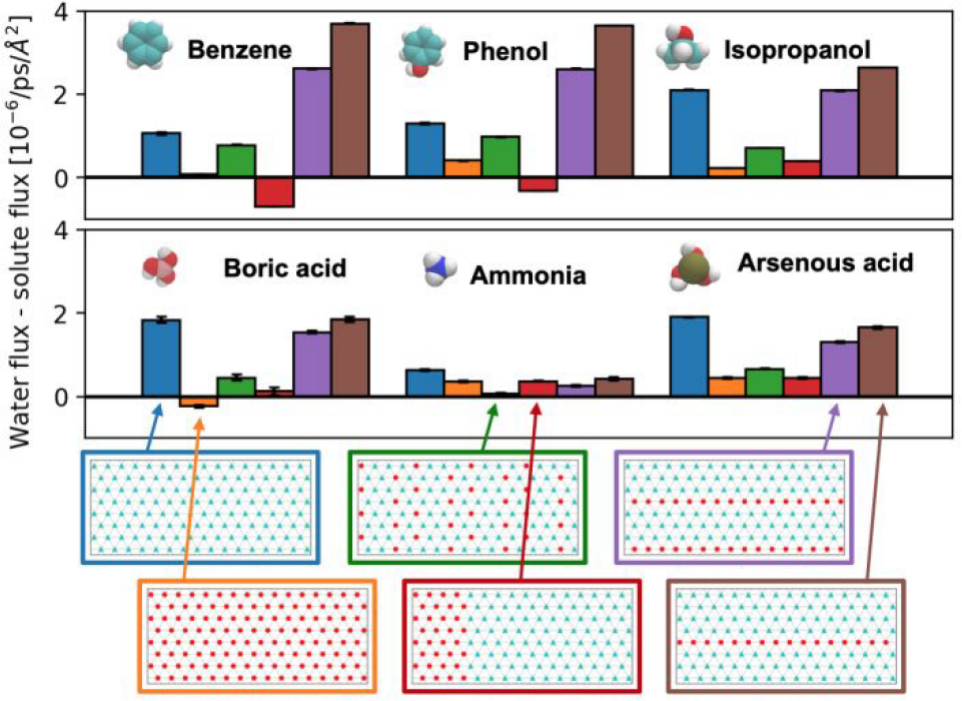
Other solutes exhibit similar trends as boric acid in a selection of rationally patterned pores. Benzene, phenol, and isopropanol show maximal water flux relative to boric acid flux in pores with rows of hydroxyl groups, while for ammonia and arsenous acid, the optimal pattern is the fully methylated one. For the simulations with solutes besides boric acid, we use the same workflow as described for boric acid except that the production simulations in the infinite pore are run for 1 μs. The reported uncertainties are the standard error of the mean from three independent simulations.

To further demonstrate the generality of these pattern motifs, we choose a diverse set of other neutral solutes (phenol, benzene, isopropanol, ammonia, arsenous acid) to simulate with several exemplar patterns. These solutes possess a wide range of affinities to methylated and hydroxylated self-assembled monolayers (measured in ref (5)) as well as a diverse set of surface hydrogen-bonding behaviors, as quantified by the average number of hydrogen bonds formed with the fully hydroxylated pore (Table S6). Out of these solutes, phenol, benzene, and isopropanol similarly demonstrate enhanced water flux relative to boric acid flux in pores with rings of hydroxyl groups (Figure 4), suggesting that the proposed mechanism may be broadly generalized to enhance the separation of other solutes from water. Consistent with the proposed diffusive hopping mechanism, there is a correlation between the enhancement in solute rejection in ringed pores and the difference in affinity for methylated versus hydroxylated SAMs (Table S6), as larger differences in affinity raise the free energy barrier to move between ring-separated methylated sections. For instance, ammonia, which has the lowest difference in affinities for the methylated and hydroxylated surfaces, has the lowest flux relative to water in the fully methylated pore. Measures of affinity to homogeneous functionalized surfaces are thus predictive of this effect of patterned functionalizations on solute rejection.

## Conclusions

The present simulations demonstrate that pore wall patterning of nonpolar and polar functional groups significantly affects the partitioning and transport of water and solutes and offers new strategies to design pore chemistries. We show that inverse design algorithms automatically identify unique, nonintuitive chemical patterning strategies and associated mechanisms that decouple and thus optimize the flux of water relative to solutes of interest. Here, for example, we find that pores with higher rates of boric acid rejection contain rings of hydroxyl groups. The underlying mechanism identified by the evolutionary optimization is unexpected: boric acid transport in optimal pores is well described by a periodic barrier-hopping process, whereby it adsorbs at the methylated regions of the pore wall and then must hop over hydrogen-bonding hydroxyl rings. These results suggest that leveraging functional groups to create a fluctuating free energy landscape for a solute in a pore may be an important mechanism for enhancing solute rejection at little cost to water permeability. More generally, this approach illustrates the use of computational inverse design algorithms to design membranes and other interfacial systems to identify unique, nonobvious solutions that can then suggest theoretical models for guiding design strategies.

Our specific results imply that as synthetic advances make multifunctional membrane surfaces more accessible, inverse design procedures will be key to exploring the larger design space. While direct functionalization of membrane surfaces with precisely patterned methyl and hydroxyl groups may not be currently feasible, lithographic techniques with block copolymer assembly as well as covalent or noncovalent functionalization of nanochannels with biomolecules or polymers^36,53,54^ may be promising routes to introducing multiple chemistries at a pore surface. The actual design space therefore potentially includes a large set of possible functionalities, much larger than the two functionalities examined here. Beyond multifunctional pore walls, other possible extensions of this pore design problem include (1) varying the pore radius, as the difference in solute and water affinity for the pore wall may offer a means to enhance water flux in the bulk region of the pore while still relying on the pore wall to enhance solute rejection, and (2) varying the topology or “roughness” of the pore surface, which has been shown to modulate local water behavior. These expanded design spaces make the development of automated design algorithms even more important. Beyond membranes, workflows for the precise and efficient *in silico* design of chemical patterns would be useful for identifying optimal antifouling surfaces and directing biomolecular folding and assembly, among other applications.

## Methods

### Simulation Models

To model an ideal nanopore with tunable pore wall chemistry, we simulate a (16,0) zigzag single-wall carbon nanotube (CNT) with nonpolar methyl and polar hydroxyl functional groups directly tethered to the interior of the pore. At smaller radii, the diffusivity of water inside the pore decreases rapidly due to strong confinement effects (Figure S1), consistent with experimental work on water in porous silica showing an enhancement in water network structures with decreasing pore size.^55,56^ We thus choose a slightly larger radius of 1.25 nm, for which water reaches its bulk density in the middle of the pore (Figure S2), in order to focus on the effects of surface chemistry as opposed to confinement, since confinement can significantly alter water and solute behavior in nanoporous environments.^57−59^ The functional groups are placed every 4 carbons (Figure S3) to achieve a surface density of 0.21 nm^2^/group, comparable to the surface density for realizable functionalized surfaces, such as self-assembled monolayers.^60^ We obtain nonbonded force field parameters for the CNT and functional groups from ref (61). Section S1C, Supporting Information, describes the bonded parameters in more detail. Parameters for water are from the TIP3P water model,^62^ and those for boric acid are based on the classical model parametrized in ref (63) (section S1CII, Supporting Information). Section S5E, Supporting Information, describes the parameters for the other solutes examined.

To compute the transport properties of water and boric acid through the pore, we simulate an “infinitely” long nanopore (Figure 1b) with 16 rows of carbon atoms, yielding a pore of length 3.4 nm with 128 functional groups, allowing a diverse set of patterns. To compute water and solute densities inside the pore, we first simulate a “finite” pore in between two water reservoirs bounded by graphene sheets (Figure 1b, details in section S1, Supporting Information). We model all systems using the OpenMM simulation engine.^64^ Electrostatic interactions are computed using particle-mesh Ewald. Lennard–Jones (LJ) nonbonded interactions are cut off at 10 Å. Hydrogen bonds are constrained with SHAKE,^65^ and water is kept rigid with SETTLE.^66^ We perform an energy minimization before all simulations. We use Langevin dynamics with a time step of 2 fs, a temperature of 298 K, and a friction coefficient of 1 ps^–1^. For NPT simulations, we use an anisotropic Monte Carlo (MC) barostat that only adjusts the box size in the axial direction, with 200 fs between MC moves to set the pressure to 1 bar. All carbon atoms in the nanotube and graphene sheets are fixed during the simulation. More details, including the lengths of the equilibration and production stages for each simulation, are given in section S1D, Supporting Information.

### Finite Nanopore Simulation and Partition Coefficient Calculation

For all simulations with the finite pore, we restrain the boric acid molecule so that it stays within the nanopore (section S1E, Supporting Information). To determine the partition coefficient of boric acid, we first compute its free energy of solvation inside the pore via an expanded ensemble simulation where the boric acid LJ ε parameters and partial charges are slowly turned on in several distinct states. The procedure is similar to that in ref (5) except that an additional restraint keeps boric acid inside the pore (section S1E, Supporting Information). The free energy of solvating boric acid, Δ*G*, is the difference between the states in which the boric acid molecule is fully on and off, which we compute using MBAR.^67,68^ We then compute the partition coefficient, *K*, from *K* = *K*_0_ exp[−(Δ*G* – Δ*G*_0_)], where Δ*G*_0_ = −3.4*k*_B_*T* and *K*_0_ = 8.0 are reference values computed from an unbiased simulation of the fully methylated pore (section S1F, Supporting Information).

### Infinite Nanopore Simulation and Transport Metric Calculations

To probe the diffusivity of water and boric acid, we compute the axial mean squared displacement for the water oxygens and boron atom and extract the diffusivities from the slope of the MSDs. To compute the nonequilibrium flux of water and boric acid under an applied pressure gradient, we add a constant acceleration to each water and boric acid atom such that the pressure gradient is 12 MPa/nm. In this regime, water flux remains linear with pressure gradient (Figure S9), allowing extrapolation to lower, more realistic gradients. We run a simulation with the applied pressure gradient and compute the total net displacements of the water oxygens and boron atoms in the axial direction. The in-pore flux is the displacement divided by the length of the simulation, the volume of the pore, and the number of molecules. This “flux” calculation measures the rate at which individual water or solute molecules move through the pore and does not have a direct contribution from their densities in the pore, which is convenient for separating the effects of pore wall chemistry on partitioning into the pore and transporting through the pore. To obtain metrics that combine both effects, we compute the permeability by multiplying the partition coefficient by the computed in-pore flux, pore volume, and bulk water density and dividing by the pressure gradient. We compute the selectivity from the ratio of the product of the partition coefficient and in-pore flux for water and boric acid and then divide by the approximate mole fraction of boric acid in seawater,^21^ approximately 1.4 × 10^–6^.

### Genetic Algorithm Structure

The genetic algorithm, depicted in Figure 1c and in more detail in Figure S10, follows the general procedure established in ref (21) but is used here to find the arrangement of 128 hydroxyl and methyl groups that optimizes the difference between water and boric acid flux. We perform two genetic algorithm runs, one in which the number of surface hydroxyl groups is fixed at 32 (“fixed fraction”) and one in which it is free to vary (“varying fraction”). Both runs begin with 5 generations of randomly generated patterns. For the fixed fraction run, we generate initial patterns by randomly choosing 32 sites for the hydroxyl groups. For the varying fraction run, the number of hydroxyls is randomly chosen at a given hydroxyl fraction spanning 0–1. We then quench these initial randomly generated patterns to minimize the pattern entropy, creating a more feature-diverse set of motifs (Figure S13) to initialize the algorithm, improving its convergence. Here, the pattern entropy is , where *P*_*i*_ gives the probability (fraction) of 3 × 3 subsections with a given pattern among all 512 distinct 3 × 3 motifs (see section S2A, Supporting Information, for details).

In the genetic optimization runs, the initial “random” generations are followed by a subsequent set of maximization generations where the flux difference is evaluated from molecular dynamics (MD) simulations, as described above. Because the simulation–evaluated flux differences are computationally expensive, we fit a surrogate model to the initial simulated generations to predict the flux difference based on a linear combination of 22 pattern features, chosen to elucidate various spatial correlations in the arrangement of the functional groups (section S3A, Supporting Information). We then rapidly iterate over many generations using the surrogate model, followed by additional generations with explicit MD evaluation of the best candidates, and finally additional surrogate-model-evaluated generations with a retrained surrogate model. Table S3 gives the number of generations in each stage. Because the varying hydroxyl group fraction case explores a much larger pattern space, we increase the number of generations compared to the fixed fraction run.

## Supporting Information Available

Details regarding the simulation models and property calculations; genetic algorithm workflow; surrogate function; spectral clustering of the genetic algorithm-identified patterns; types of rationally designed patterns explored; other quantities computed for the rationally designed patterns including the partition coefficients of water and boric acid; analytical model describing boric acid diffusivity; details regarding the simulation of other solutes in the nanopore; permeability–selectivity behavior of random and genetic-algorithm-discovered patterns (PDF)
