# Supplementary material for: Inverse Design of Pore Wall Chemistry To Control Solute Transport and Selectivity
Source: ACS Cent Sci. 2022 Nov 30;8(12):1609–17. doi: 10.1021/acscentsci.2c01011 (PMC9801506; doi:10.1021/acscentsci.2c01011)
Supplement: Supplementary file 1 — oc2c01011_si_001.pdf [file oc2c01011_si_001.pdf]

Supporting Information: Inverse design of pore wall chemistry to control solute transport and selectivity

Sally Jiao[1], Lynn E. Katz[2], M. Scott Shell[1]*

[1]Department of Chemical Engineering, University of California, Santa Barbara, California, 93106 U.S.A.
[2]Department of Civil, Architectural and Environmental Engineering, University of Texas at Austin, Austin, TX 78712

*Corresponding author. Email: shell@ucsb.edu

Table of Contents

S1. Simulation models and property calculations *3*

S1A. Pore geometry 3

S1B. Functional group layout 4

S1C. Forcefield details 4

S1CI. Bonded parameters 4

S1CII. Boric acid forcefield 5

S1D. Overview of pore simulation steps 6

S1E. Finite pore setup 7

S1F. Partition coefficient calculation 7

S1G. Infinite pore setup 8

S1H. Flux calculation 8

S2. Genetic algorithm workflow *10*

S2A. Pattern entropy minimization 10

S2B. Maximization generations 12

S2C. Model-driven generations 12

S2D. Generations in each stage 13

S3. Surrogate function *14*

S3A. Features 14

S3B. Surrogate model performance 15

S3C. Alternative surrogate models 15

S3D. Feature importance 16

S4. Pattern clustering *17*

S5. Rationally designed patterns *18*

S5A. Patterns and computed transport properties 18

S5B. Diffusivity of water and boric acid 20

S5C. Transport of water versus boric acid 21

S5D. Analytical model for boric acid transport 21

S5E. Other solutes 22

S5F. Partition coefficients 23

S6. Permeability-selectivity trade-off *24*

References *26*

S1. Simulation models and property calculations

S1A. Pore geometry

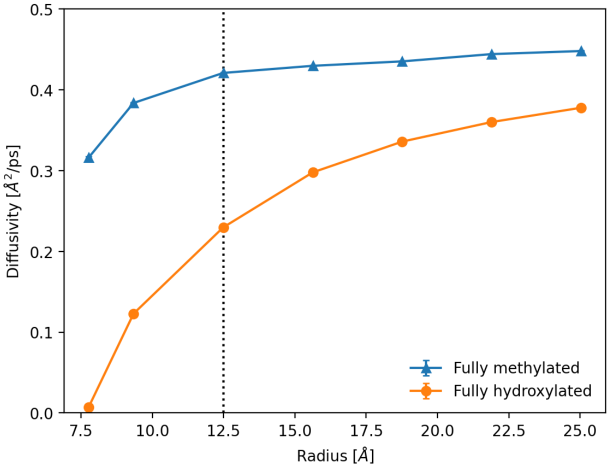

Figure S1: As the pore radius increases, water diffusivity in fully methylated (triangles) and fully hydroxylated (circles) pores increases and the difference between the diffusivity in the fully methylated and fully hydroxylated pores shrinks. The dotted, black line marks the pore radius used in the rest of this work. We use the same workflow described in the main text to simulate these pores and measure the diffusivity and uncertainty (smaller than the markers in this plot) but omit the boric acid solute.

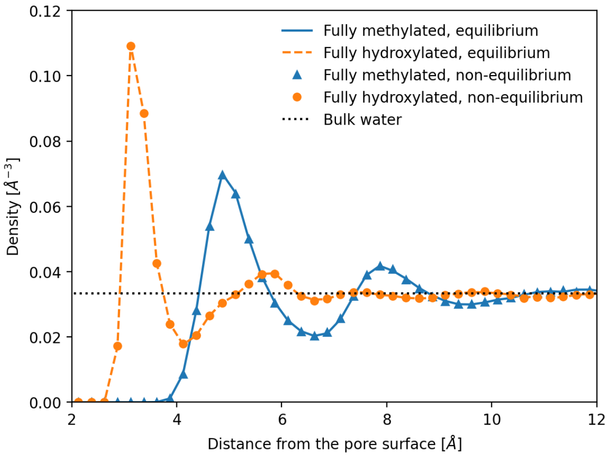

Figure S2: The radial density profiles illustrate that at a pore radius of 1.25 nm, the water density reaches that of the bulk in the middle of the pore, but the pore wall chemical functionality has a significant effect on water structuring inside the pore for the fully methylated (blue) and fully hydroxylated pores (orange). The profiles from the equilibrium (lines) and nonequilibrium (markers) simulations are consistent, suggesting that the applied pressure gradient is not large enough to perturb water structure from equilibrium. We use the same workflow described in the main text to simulate these pores but omit the boric acid solute.

S1B. Functional group layout

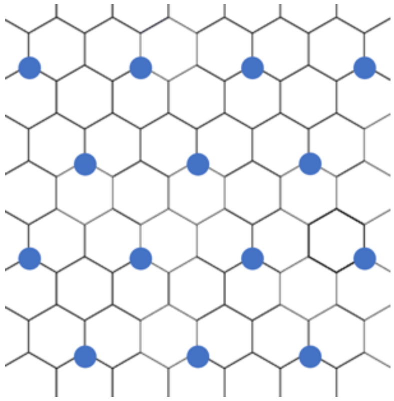

Figure S3: A schematic showing how functional groups (blue circles) are arranged on the carbon nanotube lattice. The functional groups are placed every 4 carbons to achieve a surface density of 0.21 $nm^2/group$.

S1C. Forcefield details

S1CI. Bonded parameters

The CNT carbon-carbon bond lengths are 0.142 nm. Harmonic bond and angle parameters for the functional groups are taken from the OPLS-AA/L forcefield[1] as described in Tables S1-S2. Angle potentials and dihedral potentials between the functional group and carbon nanotube atoms are set to 0. All 1-3 LJ and electrostatic interactions are set to 0. 1-4 LJ interactions between the carbon nanotube atoms and functional group heavy atom are computed using the Lorentz-Berthelot combining rules, while 1-4 LJ interactions between the carbon nanotube atoms and functional group hydrogens are set to 0. 1-4 electrostatic interactions are multiplied by 0.8333. This keeps the functional groups inside the nanotube while allowing them to rotate freely.

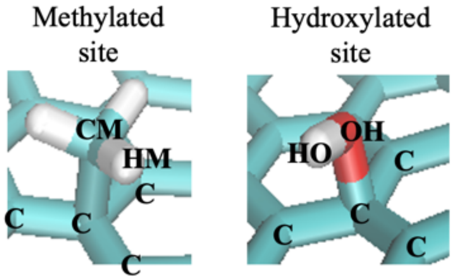

Figure S4: Labeling of atoms in methylated and hydroxylated site for Tables S1-S2.

Bond	b_0 $[nm]$	K_b $[kJ/mol/nm^2]$	Source
C-CM	0.1529	224262.4	OPLS-AA/L CT-CT
CM-HM	0.109	284512	OPLS-AA/L CT-HC
C-OH	0.141	267776	OPLS-AA/L CT-OH
OH-HO	0.0945	462750.4	OPLS-AA/L OH-HO

Table S1: Harmonic bond parameters for functional groups.

Angle	θ_0 $[deg]$	$K_\theta[kJ/mol/rad^2]$	Source
C-C-CM	0	0	--
C-C-OH	0	0	--
C-CM-HM	110.7	313.8	OPLS-AA/L CT-CT-HC
HM-CM-HM	107.8	276.144	OPLS-AA/L HC-CT-HC
C-OH-HO	108.5	460.240	OPLS-AA/L CT-OH-HO

Table S2: Harmonic angle parameters for functional groups.

S1CII. Boric acid forcefield

Simulations with the boric acid dihedral potential (H-O-B-O) from Ref. [2] result in long rotation times around the oxygen-boron bond, resulting in poor sampling of different dihedral states (Fig. S5). For this work, we reduce the force constant by half to enhance sampling (Fig. S5) in lieu of running separate simulations for both dihedral states. The force constant remains significant (over $3k_BT$ even after reduction, and thus still enforces planarity in the molecule. This reduction does not affect the transport properties (Fig. S6).

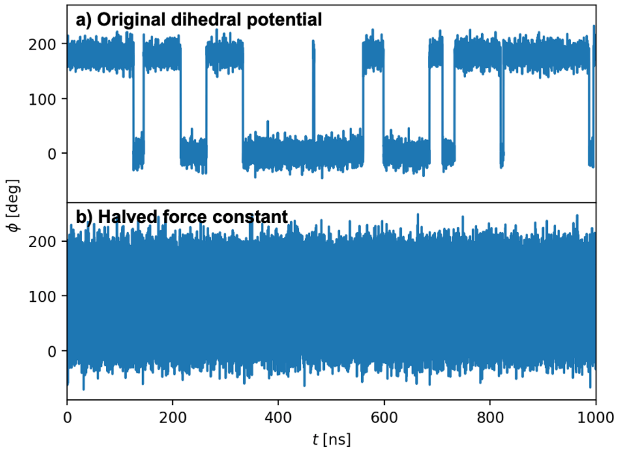

Figure S5: Boric acid dihedral (H-O-B-O) angle with (a) the original dihedral potential from Ref. [2] and (b) the force constant halved. With the original potential, isomerization of the dihedral occurs on the order of hundreds of ns, resulting in poor sampling of the dihedral states in the 100-ns production simulation used by the genetic algorithm to compute boric acid flux. Reducing the force constant significantly enhances the sampling of dihedral states. The workflow is the same as described in the main text for the infinite pore system with applied pressure gradient, except that the production simulations are run for 1 μs.

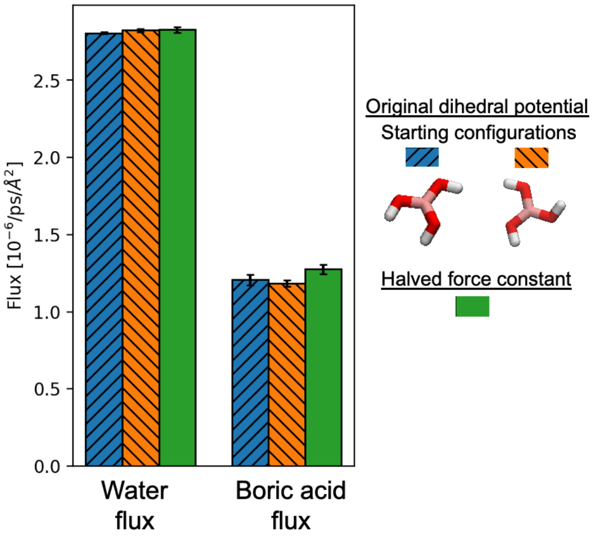

Figure S6: The measured water and boric acid flux does not change when the dihedral force constant is altered. The workflow is the same as described in the main text for the infinite pore system with applied pressure gradient, except that the production simulations are run for 1 μs. Furthermore, an additional harmonic potential with force constant 20 $kJ/mol/rad^2$ is applied to the dihedral during the initial NVT equilibrations and is set to achieve the starting configurations in the figure. The reported fluxes are computed by averaging from three independent simulations. The reported uncertainty is the standard error of the mean.

S1D. Overview of pore simulation steps

For each pattern, the following main calculations are performed:

1. **Simulate a finite pore between two reservoirs (Fig. 1b in main text) with a single boric acid molecule to compute the average water density inside the pore.** We run a 100-ps NVT equilibration, then a 10-ns NPT equilibration, and finally a 20-ns NPT production simulation.
2. **Prepare an infinite pore (Fig. 1b in main text) with the same water density as the finite pore.**
3. **Compute the flux of boric acid and water in the infinite pore under an applied pressure gradient via a nonequilibrium simulation.** We run a 2-ns NVT equilibration followed by a 100-ns NVT production simulation (in the genetic algorithm) or a 200-ns NVT production simulation (to examine the rationally designed patterns). The first 10 ns are discarded when computing properties.

For each rationally designed pattern (i.e. beyond those explored by the genetic algorithm), the following additional calculations are performed:

4. **Run an expanded ensemble simulation turning on the boric acid non-bonded parameters to compute the free energy of solvation in the pore, and thus the partition coefficient of boric acid inside the pore.** We run a 500-ps NVT equilibration and then a 500-ps NPT equilibration with all non-bonded and partial charges turned on. Subsequently, we run the expanded ensemble simulation in the NPT ensemble (500 fs between MC barostat moves) for 10 ns to equilibrate state weights and then for 410 ns with fixed weights, changing expanded ensemble states every 1 ps. We use the final 410 ns when computing properties.
5. **Compute the diffusivity of boric acid and water in the infinite pore without an applied pressure gradient via an equilibrium simulation.** We run a 2-ns NVT equilibration followed by a 1-μs NVT production simulation.

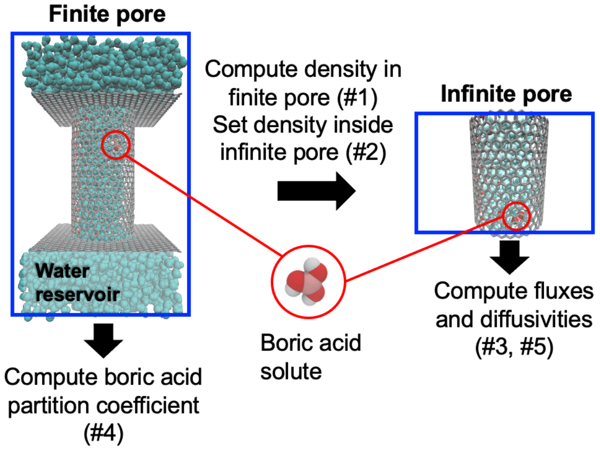

Figure S7: Visualization of the finite pore and infinite pore systems (reproduction of the snapshots from Fig. 1b in the main text at a larger scale), annotated with the steps outlined in section S1D for simulating the pores and computing the transport properties.

S1E. Finite pore setup

To eliminate end effects in the finite pore, we first repeat the pore pattern for 4 additional rows in both directions, creating a pore of length of 5.0 nm. Calculations of density and partition coefficients are performed excluding this added region.

To bound the water reservoirs, we create two graphene sheets with width and length equal to 5 nm (4 times the pore radius), with openings for the pore created by (1) deleting all carbon atoms within the convex hull defined by carbon atoms in the graphene sheets that are closest to CNT carbon atoms and then (2) bonding pairs of graphene and CNT carbon atoms that are within 0.242 nm of each other. The graphene and CNT positions are fixed during the simulation; these bonds are needed only so that the barostat recognizes them as a single molecule. Nonbonded forcefield parameters for the carbon atoms in the graphene sheet are the same as those for the CNT. We solvate the entire box using GROMACS[3] and then remove all waters in between the graphene sheets (region laterally external to the pore). We then insert a single boric acid molecule using GROMACS.

For simulations with the finite pore, unless otherwise noted, we restrain the center of mass of the single boric acid so that it remains within the nanopore using a flat-bottom harmonic restraint, $0.5\ k\ (dz - dz_0)^2 step(dz\ - dz_0)$ where dz is the distance along the pore axis between the boric acid heavy atoms' center of mass and a carbon atom near the center of the pore, $dz_0 = 1.70384\ nm$ (half the pore length, minus the added region length), $step(x) = 1$ when $x\ \geq\ 0$ and is 0 otherwise, and $k = 1000\ kJ/mol/nm^2$.

For the expanded ensemble simulation, we additionally apply a flat-bottom harmonic restraint to the boric acid solute in the radial direction to keep it inside the pore. The restraint has the form $0.5\ k_r\ (r - R)^2 step(r\ - R)$ where r is the distance in the xy-plane between the boric acid heavy atoms' center of mass and the center of the pore, R is the pore radius, $step(x) = 1$ when $x\ \geq\ 0$ and is 0 otherwise, and $k_r = 1000\ kJ/mol/nm^2$.

S1F. Partition coefficient calculation

The partition coefficients of boric acid between bulk water and the pore are computed as described in the main text. The reference values $K_0 = 8.0$ and $\Delta G_0 = -3.4\ k_B T$ are computed for the fully methylated pore, where the partition coefficient is computed from an unbiased MD simulation in the finite pore system with the same setup as described previously but with 20 boric acid molecules (placed initially in the water reservoir using GROMACS). For the unbiased MD simulation, we perform a 100-ps NVT equilibration followed by a 100-ns NPT equilibration where we save configurations every 1 ns. We set the box length in the axial direction to the average of the second half of the NPT equilibration. We then perform an additional 100-ns NVT equilibration. Finally, we perform a 400-ns NVT production. The friction coefficient for Langevin dynamics is 0.1 ps^{-1}. We do not restrain the boric acid molecules in these simulations and compute the partition coefficient as the ratio of the average boric acid density inside the pore by the average boric acid density in the water reservoir (excluding the region within 5 Å from the graphene sheets). We do

this for each 80-ns block in the production simulation and then average the resulting partition coefficients. Fig. S8 shows the agreement between the partition coefficients computed from the expanded ensemble and unbiased simulations.

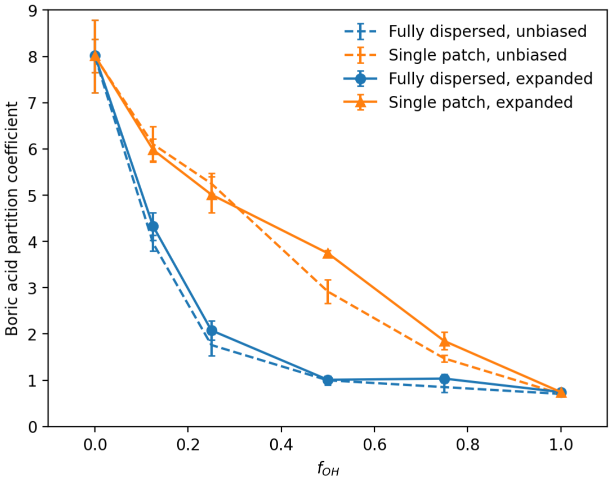

Figure S8: Partition coefficients computed from the free energy of solvating the boric acid inside the pore (solid), as described in the main text, compared to those computed from the unbiased MD simulations described above (dashed), show good agreement. For the former, three independent runs are averaged together and the uncertainty is the standard error of the mean. For the latter, the standard error of the average partition coefficient from 5 80-ns blocks, as described earlier, is reported. The orange markers represent patterns with a "single patch" of hydroxyl groups, spanning the pore axis, while the blue markers represent "fully dispersed" patterns

The partition coefficient of water computed from the expanded ensemble simulation described in the main text is the reweighted average density of water in the pore relative to the average density in the water reservoir (excluding the region within 5 Å from the graphene sheets).

S1G. Infinite pore setup

We generate the initial positions of the CNT carbons and functional groups using an in-house script. The positions of the CNT carbon atoms are then fixed during the simulation. We then place a single boric acid molecule into the pore using GROMACS. Finally, we solvate the pore by placing waters in one by one, randomly drawing their center of mass positions and orientations. We continue to adjust waters until their number in the pore equals that of a finite pore with the same pattern connected to a water reservoir (without the end regions).

S1H. Flux calculation

To compute the flux of water and boric acid under an applied pressure gradient, we add a constant acceleration to each water and boric acid atom. The added acceleration is $a = \frac{dP}{dz} R^2 L\pi / M$, where $\frac{dP}{dz}$ is the desired pressure gradient, R is the radius of the nanopore, L is the length of the nanopore, and M is the total mass of the water molecules and boric acid solute. The pressure gradient is set to $\frac{dP}{dz} = 12\ MPa/nm$, which is higher than pressure gradients in typical membranes. However, measurements of water flux over different pressure gradients reveal that $12\ MPa/nm$ is still within the range where water flux is linear with pressure gradient, suggesting that the applied force is small enough that the transport trends can be extrapolated to lower gradients.

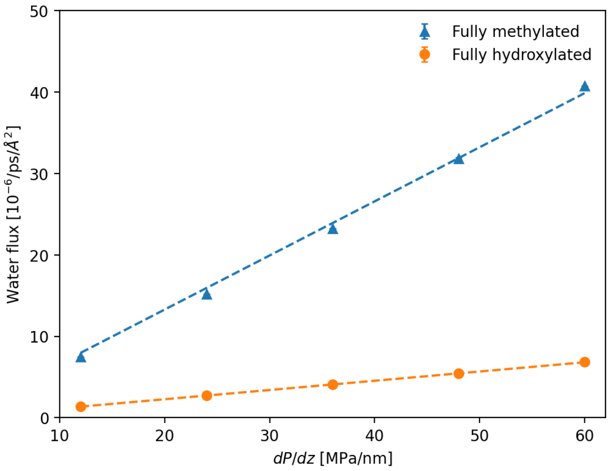

Figure S9: The average molar flux of water is linear with respect to the pressure gradient. We use the same workflow described in the main text to simulate these pores and compute the water flux and uncertainty (smaller than the markers in this plot) but omit the boric acid solute. The dashed lines show the lines of best fit, with the intercept fixed at the origin.

To compute the flux, we divide the production simulation into 5 blocks. In each block, the total net displacements of the water oxygen atom and boric acid boron atom are computed and divided by the length of the block, the volume of the pore, and the number of molecules (water or boric acid, depending on the calculation). The fluxes are averaged over the 5 blocks.

S2. Genetic algorithm workflow

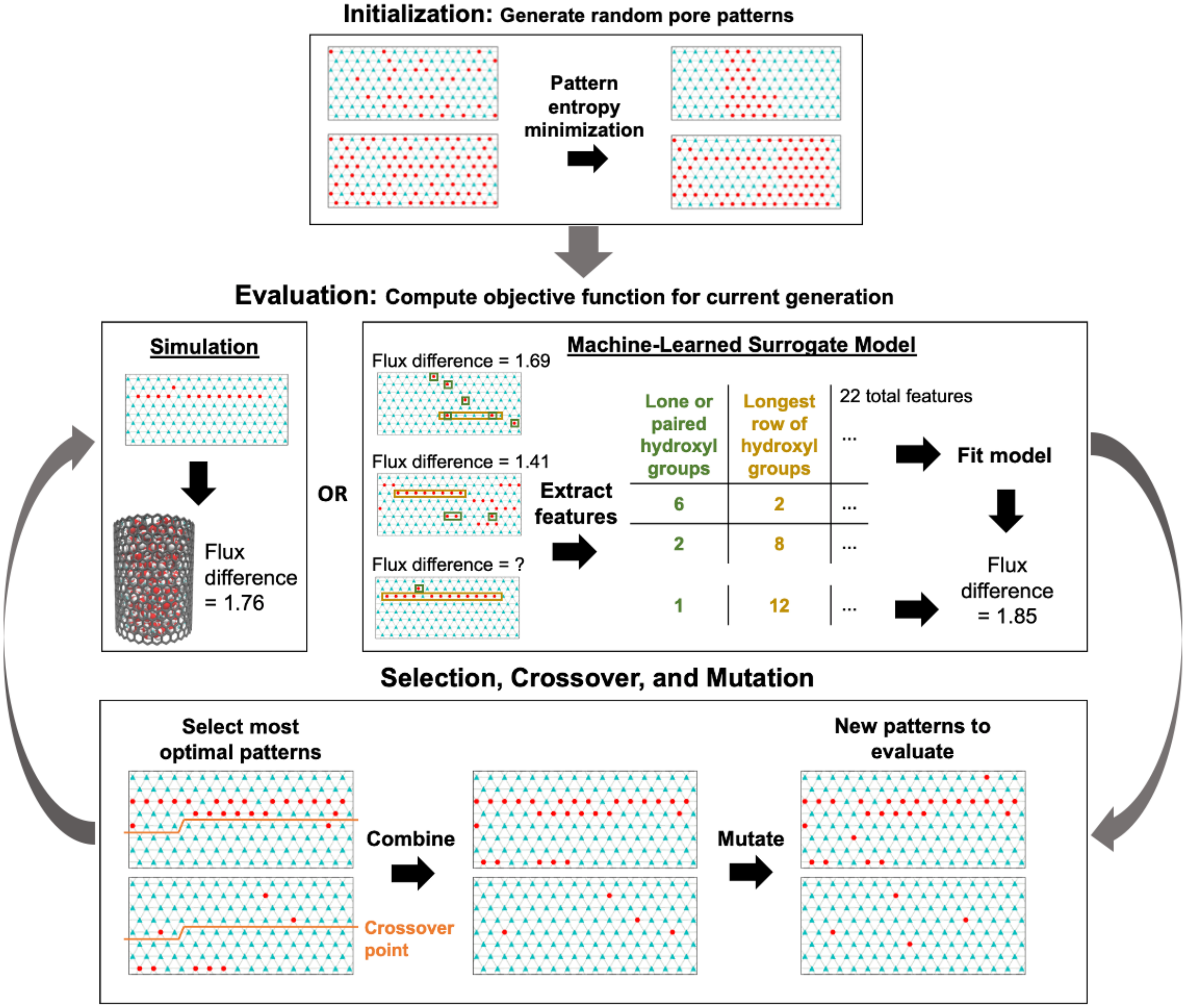

Figure S10: The genetic algorithm workflow.

S2A. Pattern entropy minimization

The pattern entropy is computed as $S = -\sum_{i=1}^{512} P_i log P_i$ where i is an index over all $2^9 = 512$ possible 3x3 arrangements of methyl and hydroxyl groups and P_i is the fraction of 3x3 subsections of the pattern (128 subsections in total) that have the ith arrangement. For instance, the index corresponding to the 3x3 arrangement in the subsections marked in the pattern in Fig. S11 is $i = 447$ and the 3x3 arrangement appears twice in the pattern, as marked in Fig. S11. Thus, $P_{447} = 2/128$.

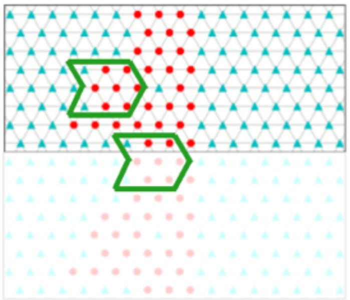

Figure S11: Example pattern with 3x3 subsections marked to illustrate pattern entropy calculation. The calculation accounts for periodic boundaries, as depicted here.

The pattern entropy is minimized via quenching. In each step of this procedure, the positions of one hydroxyl and one methyl group are exchanged. The change in pattern entropy, ΔS, is computed and the move is accepted with probability $min[1, exp(-\Delta S/T)]$ where T is a ("temperature") parameter that adjusts the rate at which moves that increase pattern entropy are accepted. For the random generations, we run this quenching procedure on each generated pattern for 1000 steps at a fixed temperature of 0.01. Fig. S12 shows example quenching trajectories. This pattern entropy minimization procedure creates more feature-diverse patterns (sampling a wider range of pattern features) as shown in Fig. S13. Later in the genetic algorithm, we also use the pattern entropy quenching minimization to refine the first generation of patterns created by the second set of MD-driven generations; this time, the algorithm is run for 10,000 steps at a temperature of 0.05.

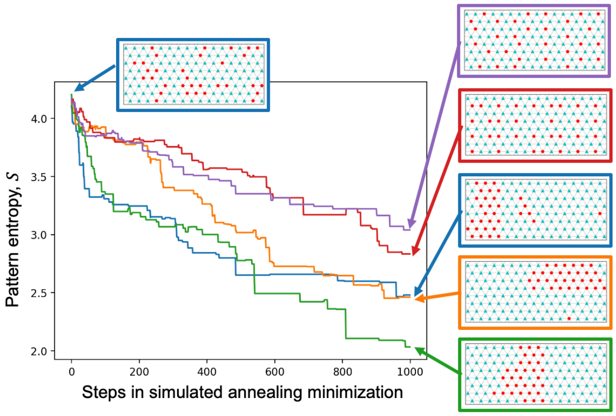

Figure S12: Example quenching trajectories. Patterns with 32 hydroxyl groups are generated by randomly choosing 32 sites. Each pattern is then entropy-minimized through the quenching procedure for 1000 steps at a temperature of 0.01.

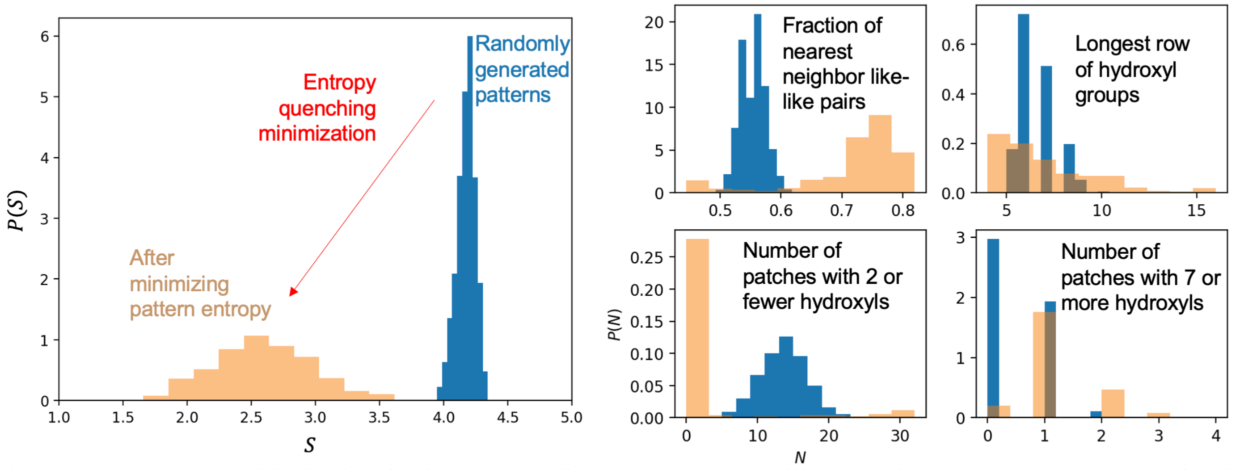

Figure S13: Entropy minimization leads to more diverse patterns. To illustrate this, we create 1000 “randomly generated” patterns by randomly choosing 32 sites to be hydroxyl groups. We also create 1000 “entropy minimized” patterns by again randomly choosing 32 sites to be hydroxyl groups and then minimizing pattern entropy by running the quenching algorithm for 1000 steps at a temperature of 0.01. (Left) The quenching algorithm leads to lower pattern entropies in the entropy minimized patterns (tan) versus the randomly generated patterns (blue). (Right) Histograms over various pattern features show that the entropy minimized patterns (tan) cover a larger range of pattern feature values compared to the randomly generated patterns (blue).

S2B. Maximization generations

In the genetic algorithm, the random generations are followed by a “maximization” generation, which follow the procedures described in Ref. [4]. Briefly, in each maximization generation, a set of 8 optimal parent patterns is chosen via a tournament selection procedure. These parent patterns are randomly grouped in pairs and combined to form new child patterns. The patterns are then mutated. The mutation rate used here is 0.04.

S2C. Model-driven generations

The only difference between the model-driven generations and the MD-simulated generations is the calculation of the flux difference. Models of the water and boric acid flux are fit based on 22 features of the pattern (section S3A lists the features used). The sklearn package is used to scale the features and the log of the water and boric acid fluxes (fitting the log of the fluxes was found to yield more accurate models). Then, using multiple linear regression in sklearn, linear models are fit that predict the scaled logarithm of the water and boric acid fluxes from the set of pattern features. These models are then used in the model-driven generations to predict the flux difference. When the model is refit based on the second set of simulated generations, we recompute the predictions from the previous model-predicted maximization generations using the new model.

We hypothesize that the surrogate model enhances the efficiency of the genetic algorithm by making it more robust to uncertainty in the MD simulation. To demonstrate this, we test a mock objective function that is the “correlation” with an “ideal” pattern. Here, the correlation is the number of functional groups of the same type in the same position (e.g. the maximum overlap is 128) and accounts for all translations and reflections of the pattern. Since the correlation can be computed quickly (in contrast to an MD simulation), this mock objective function provides a way to rapidly test the genetic algorithm workflow. We can also artificially introduce “error” to the correlation calculation by adding Gaussian noise. Fig. S14 shows that when there is error in the

objective function, the genetic algorithm finds, on average, more optimal patterns when it uses the surrogate function to predict the objective function. We note that more complex models with a larger number of fitted parameters compared to the linear model may be more prone to overfitting and thus may not enhance the robustness of the genetic algorithm, one of the motivations for choosing a linear model.

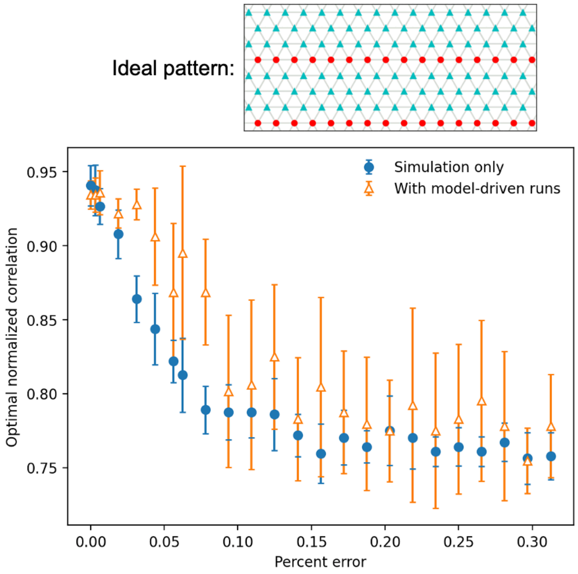

Figure S14: The genetic algorithm with the simpler objective function (the "ideal" pattern is shown at the top) is run for 200 generations. For the "simulation only" case (blue circles), the objective function evaluation is performed only by directly computing the pattern correlation and adding an error generated from a normal random variable centered at 0 with varying standard deviation, σ. For the "with model-driven runs" case (orange triangles), the algorithm begins with 20 generations where the objective function evaluation is performed as described for the "simulation only" case followed by 180 generations where the objective function is predicted from a model fit from the data from the initial 20 generations. Ten runs for both the "simulation only" and "with model-driven runs" cases are performed at varying values of σ (plotted on the x-axis normalized by 128). The optimal true correlation with an ideal surface found in each run is averaged over the 10 runs (plotted on the y-axis normalized by 128) and the error bars show the standard deviation over those 10 runs. The rest of the genetic algorithm workflow is the same as described, except that the initial random generations do not use the entropy minimization procedure. At non-zero values of error in the objective function, the runs with the surrogate model identify more optimal patterns, on average, than the runs without it.

S2D. Generations in each stage

Run	"Random"	First maximization with MD simulations	First maximization with surrogate model	Second maximization with MD simulations	Second maximization with surrogate model
Fixed hydroxyl group fraction	5	10	1000	15	1000
Varying hydroxyl group fraction	5	18	1000	18	2000

Table S3: The number of generations per stage of the genetic algorithm optimization. Since the run varying hydroxyl group fraction explores a much larger portion of the pattern space, we increase the number of generations in some of the stages compared to the fixed hydroxyl group fraction run.

S3. Surrogate function

S3A. Features

The surrogate function models the flux difference as a linear function of 22 features of the pattern, listed below:

1. Fraction of nearest neighbors of a functional group that are the same type of group. The nearest neighbors of a functional group are those surrounding it in the hexagonal lattice. Each functional group has 6 nearest neighbors.
2. Fraction of nearest neighbors of hydroxyl groups that are also hydroxyl groups
3. Fraction of nearest neighbors of methyl groups that are also methyl groups
4. Maximum number of hydroxyl groups in a row
5. Maximum number of methyl groups in a row
6. Number of patches comprising 1 or 2 hydroxyl groups. Two hydroxyl groups are in the same patch if they are nearest neighbors.
7. Number of patches of 3, 4, 5, or 6 hydroxyl groups
8. Number of patches of 7 or more hydroxyl groups
9. Number of times two hydroxyl groups are within 1 spacing apart along the pore circumference. Here, a spacing is the distance between two functional groups in hexagonal lattice.
10. Number of times two hydroxyl groups are between 1 and 2 spacings apart along the pore circumference
11. Number of times two hydroxyl groups are between 2 and 3 spacings apart along the pore circumference
12. Number of times two hydroxyl groups are between 3 and 4 spacings apart along the pore circumference
13. Number of times two hydroxyl groups are between 4 and 5 spacings apart along the pore circumference
14. Number of times two hydroxyl groups are between 5 and 6 spacings apart along the pore circumference
15. Number of times two hydroxyl groups are between 6 and 7 spacings apart along the pore circumference
16. Number of times two hydroxyl groups are within 1 spacing apart along the pore axis.
17. Number of times two hydroxyl groups are between 1 and 2 spacings apart along the pore axis.
18. Number of times two hydroxyl groups are between 2 and 3 spacings apart along the pore axis.
19. Number of times two hydroxyl groups are within 1 spacing apart.
20. Number of times two hydroxyl groups are between 1 and 2 spacings apart.
21. Number of times two hydroxyl groups are between 2 and 3 spacings apart.
22. Pattern entropy (calculation described in section S2A).

S3B. Surrogate model performance

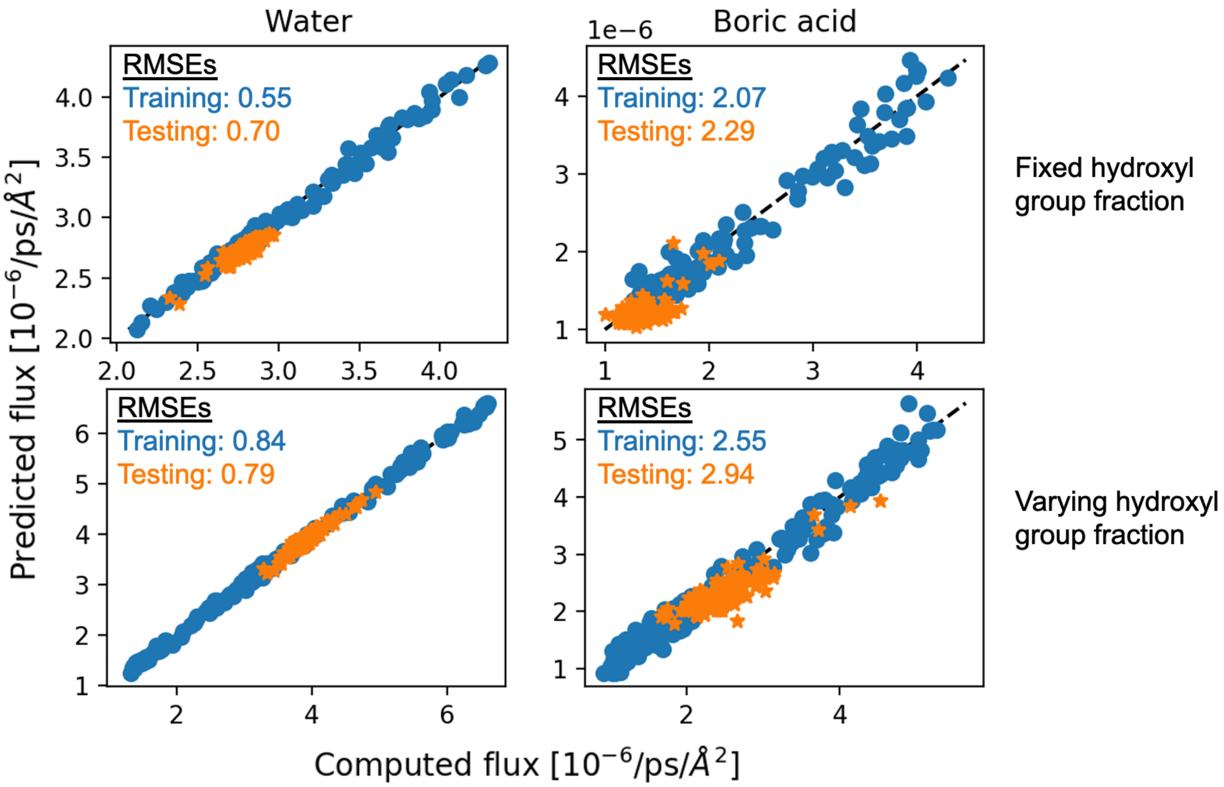

Figure S15: The surrogate model reasonably predicts water and boric acid fluxes when trained on data from the first set of simulated generations (120 patterns for fixed hydroxyl group fraction, 184 patterns for varying hydroxyl group fraction) and tested on the second set of simulated generations (120 patterns for fixed hydroxyl group fraction, 144 patterns for varying hydroxyl group fraction).

S3C. Alternative surrogate models

We compare the model used here (linear, with the set of 22 pattern features as predictors) with alternative surrogate models: LASSO (predictors: pattern features), fully connected artificial neural network (predictors: pattern features), convolutional neural network (predictors: explicit patterns), fully connected artificial neural network (predictors: explicit patterns). We use the dataset generated from the genetic algorithm run fixing hydroxyl group fraction (240 patterns and MD-computed flux differences). Instead of predicting the log of water and boric acid fluxes separately, we perform a single prediction of the flux difference. We use the sklearn package for the models where the predictors are the feature set and keras when the predictors are the patterns. For the latter, we augment the dataset with translational variants of each pattern (128 translational variants per original pattern).

For the LASSO regression, we use a regularization parameter of $\alpha = 0.04$. The artificial neural network (ANN) with feature predictors uses two fully connected layers with 25 nodes per layer. The convolutional neural network (CNN) uses 1 convolutional layer with 4 5x5 filters, 1 dropout layer with a dropout rate of 0.5, and 2 fully connected layers with 10 nodes per layer. The ANN model with pattern predictors uses 4 fully connected layers with 50 nodes per layer.

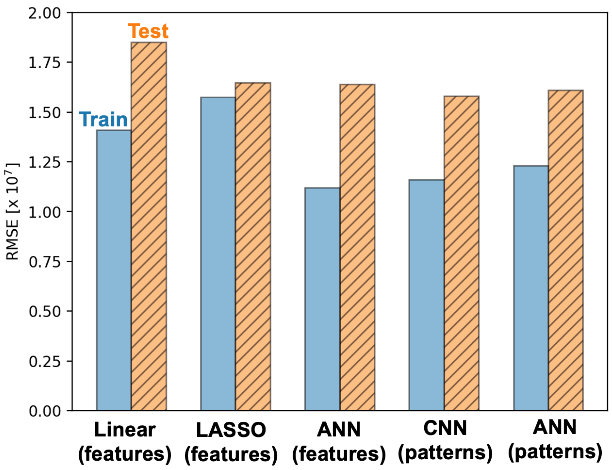

Figure S16: Comparison of alternative surrogate models shows that performance of neural-network-based models is approximately equal to that of simpler, regularized linear models. For each model, the bars show the average RMSEs computed from 5-fold cross-validation of the training (solid) and testing (hatched) sets.

S3D. Feature importance

We use all data from the simulated generations of the fixed hydroxyl group fraction run (240 patterns in total) to evaluate feature importance. We identify the most important features two different ways: (1) random forest regression and (2) LASSO regression. For both, we randomly divide the dataset into 5 different folds (48 patterns per fold), and run the regression 5 times, each time removing a different fold from the dataset and using the remaining data to fit the models. For random forest regression, we use the sklearn package to fit a random forest regressor and compute feature importance values. Features with an importance over 0.01 are listed in Table S4. For LASSO regression, we use the sklearn package to fit a LASSO model with $\alpha = 0.05$.

Fit	1	2	3	4	5
Random forest regression, features with importance over 0.01	**4**, **9**, **16**, **17**, **18**, **22**	**4**, **9**, 13, **16**, **17**, **18**, 20, **22**	**4**, **9**, 10, **16**, **17**, **18**, 19, **22**	**4**, **9**, **16**, **17**, **18**, **22**	**4**, **9**, 13, 15, **16**, **17**, **18**, **22**
LASSO regression, non-zeroed features	**4**, **5**, **9**, 11, **16**, 21	**4**, **5**, 8, **9**, 15, **16**, 18	**4**, **5**, 8, **9**, 11, 15, **16**, 18, 21	**4**, **5**, **9**, 11, **16**	**4**, **5**, **9**, **16**, 17, 18, 21

Table S4: Important features, as identified by random forest regression and LASSO regression, for each fit (a different, random fold of the dataset is removed for each fit). Feature numbers correspond to the list in section S3A. Bolded numbers are those that appear for each fit for a given regression method.

S4. Pattern clustering

We perform spectral clustering of the 240 patterns from the simulated generations of the fixed hydroxyl group fraction run. We pre-compute a 240x240 correlation matrix for all patterns where the correlation between two patterns is the number of hydroxyl groups in the same position (accounting for all translations and reflections of the pattern). We then cluster the patterns using sklearn's spectral clustering implementation, fixing the number of clusters as 6.

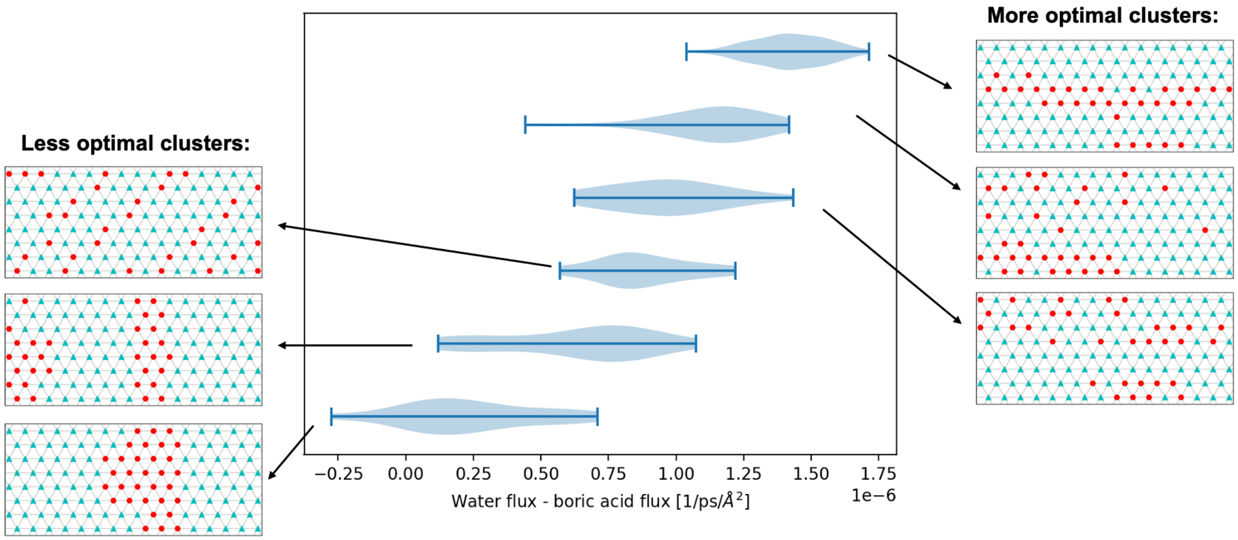

Figure S17: Spectral clustering of the 240 simulated patterns from the genetic algorithm run with fixed hydroxyl group fraction suggests that patterns with rows of hydroxyl groups and axial patches of hydroxyl groups may maximize and minimize, respectively, the difference between water flux and boric acid flux. Each violin plot represents a separate cluster. One pattern snapshot is shown for each cluster.

S5. Rationally designed patterns

S5A. Patterns and computed transport properties

Pattern	f_{OH}	K_{water}	$K_{boric\ acid}$	D_{water}	$D_{boric\ acid}$	Water flux $[10^{-6}/ps/Å^2]$	Boric acid flux $[10^{-6}/ps/Å^2]$	Water flux – boric acid flux $[10^{-6}/ps/Å^2]$
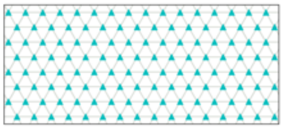	0	0.4940_1	8.0_4	0.421_1	0.24_2	7.61_2	5.76_6	1.85_8
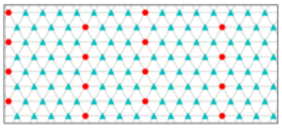	0.125	0.5310_3	4.3_3	0.3192_4	0.17_1	3.44_2	2.71_5	0.73_7
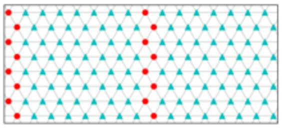	0.125	0.5234_2	5.5_4	0.350_1	0.199_9	4.57_2	3.96_3	0.61_4
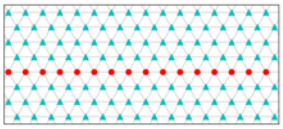	0.125	0.5234_2	5.7_2	0.357_1	0.121_2	4.44_2	2.58_8	1.86_7
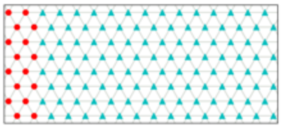	0.125	0.5205_2	6.0_2	0.3676_6	0.241_9	5.385_5	4.69_6	0.69_6
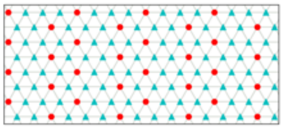	0.25	0.5652_2	2.1_2	0.2528_6	0.100_6	1.970_6	1.51_7	0.46_7
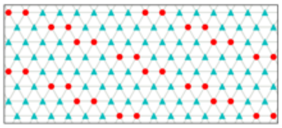	0.25	0.5574_2	3.2_1	0.2822_6	0.103_7	2.454_8	1.55_8	0.91_9
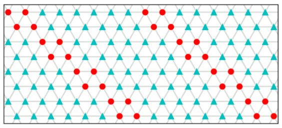	0.25	0.5500_2	4.2_1	0.307_1	0.131_5	3.191_6	2.33_1	0.86_2
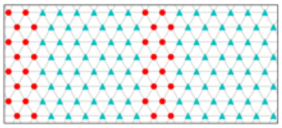	0.25	0.5469_2	4.7_1	0.3232_4	0.216_8	3.71_1	3.58_6	0.13_6
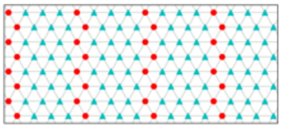	0.25	0.55259_8	3.8_2	0.2939_6	0.17_1	2.79_3	2.47_5	0.32_5

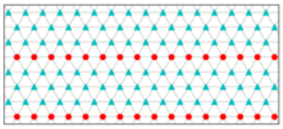	0.25	0.5530_2	4.03_9	0.299_1	0.081_5	2.83_2	1.27_3	1.55_4
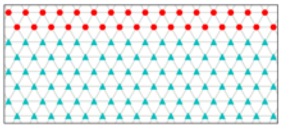	0.25	0.5507_3	4.92_8	0.3255_8	0.065_4	3.45_2	1.61_4	1.84_6
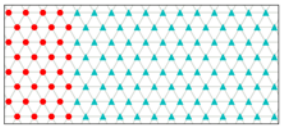	0.25	0.5454_2	5.0_4	0.3404_9	0.22_1	4.39_3	4.3_1	0.13_9
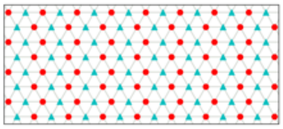	0.5	0.60762_7	1.01_6	0.2206_6	0.092_4	1.489_3	1.24_6	0.25_6
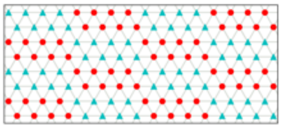	0.5	0.6054_1	1.89_7	0.2415_5	0.068_4	1.70_1	0.89_3	0.82_4
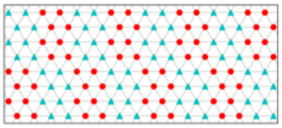	0.5	0.60115_6	2.1_2	0.2436_9	0.096_4	1.796_5	1.265_2	0.532_9
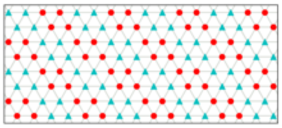	0.5	0.6041_2	1.91_7	0.2358_7	0.073_4	1.657_9	0.99_6	0.66_6
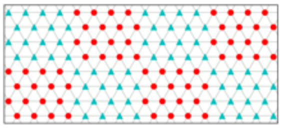	0.5	0.60085_6	2.7_2	0.257_1	0.077_5	1.967_2	1.10_4	0.86_4
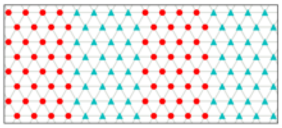	0.5	0.5961_3	2.7_2	0.2762_4	0.191_3	2.39_2	2.56_3	-0.18_5
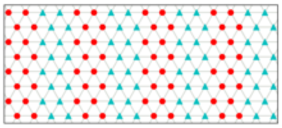	0.5	0.5989_4	1.8_2	0.2474_5	0.150_7	1.878_4	1.75_2	0.13_3
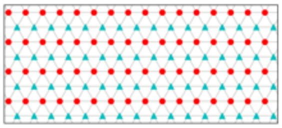	0.5	0.6084_2	1.31_7	0.2297_8	0.105_7	1.576_6	1.16_2	0.41_2
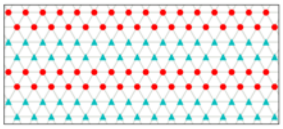	0.5	0.6061_2	2.22_8	0.2533_5	0.061_4	1.85_1	0.83_1	1.010_2

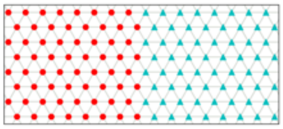	0.5	0.5943_2	3.74_7	0.2884_8	0.22_1	2.87_1	3.52_8	-0.65_8
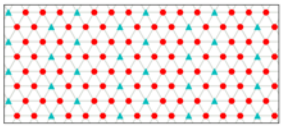	0.75	0.6553_1	1.03_9	0.2125_7	0.091_6	1.302_4	1.17_2	0.14_2
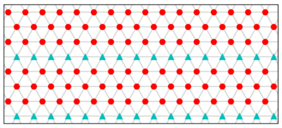	0.75	0.6534_4	1.01_1	0.2265_4	0.095_5	1.388_6	1.12_4	0.27_4
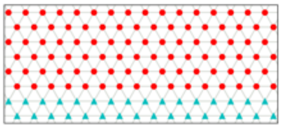	0.75	0.6484_2	1.27_5	0.2420_6	0.071_5	1.582_7	1.04_1	0.54_1
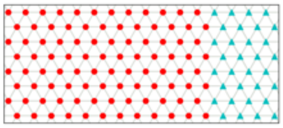	0.75	0.64309_5	1.8_2	0.2503_8	0.16_1	1.793_6	2.16_4	-0.37_4
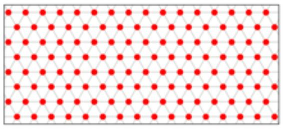	1	0.6902_3	0.743_7	0.2297_4	0.109_5	1.337_7	1.56_4	-0.23_3

Table S5: List of all rationally designed patterns and computed transport properties.

S5B. Diffusivity of water and boric acid

To compute diffusivities, we divide the 1 μs NVT production simulation into 5 blocks of 200 ns each. Within each block, we compute the mean squared displacement trajectories of the water oxygens and boron atoms over 1 ns intervals. We fit a line to the region above 100 ps and compute the diffusivity as half of the slope. We take the mean of the diffusivities fit for each of the 5 blocks to get the reported diffusivity. The reported uncertainty is the standard error of the mean.

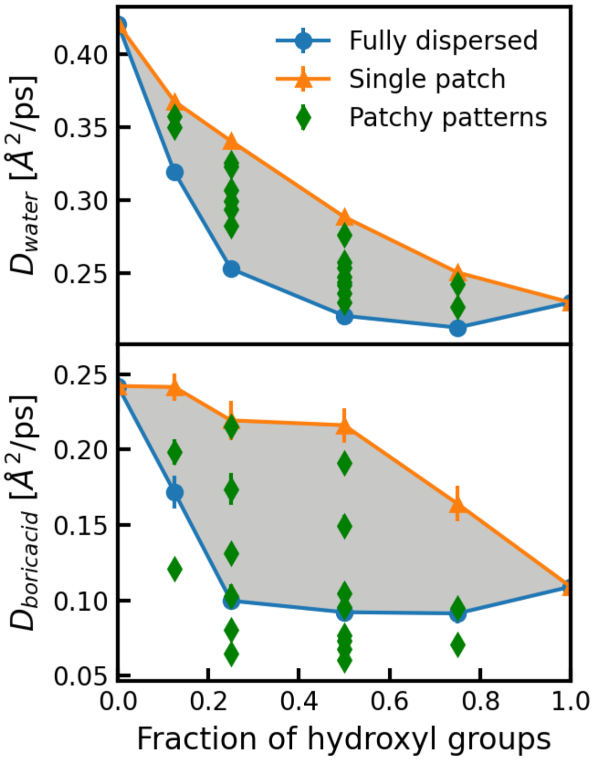

Figure S18: Diffusivities of (top) water and (bottom) boric acid for all rationally designed patterns. Note that some rationally designed patterns give lower boric acid diffusivities compared to the fully dispersed surfaces, in contrast to the case for water, producing differential effects on the behavior of boric acid relative to water.

S5C. Transport of water versus boric acid

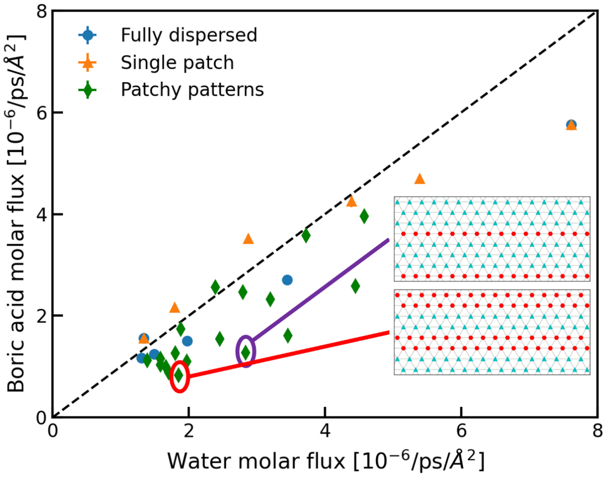

Figure S19: Unlike evenly dispersed and single-patch patterns, other non-intuitive patterns can reduce boric acid flux without affecting water flux to the same extent.

S5D. Analytical model for boric acid transport

We use the model derived in Ref. [5] for the 1-D diffusion coefficient, D, of a Brownian particle in an arbitrary periodic potential, $V(x)$, with no tilting force:

$$\frac{D}{D_0} = \frac{\int_{x_0}^{x_0+L} \frac{dx}{L} I_+^2(x) I_-(x)}{\left[\int_{x_0}^{x_0+L} \frac{dx}{L} I_+(x)\right]^3}$$

$$I_\pm(x) = \int_0^L \frac{dy}{D_0} exp([\pm V(x) \mp V(x \mp y)]/k_B T)$$

Where D_0 is the diffusion coefficient when $V(x)$ is constant and L is the period of $V(x)$, so that $V(x) = V(x + L)$.

For simplicity, we model $V(x)$ as a stepwise function, as depicted in Fig. S20, taking on a constant value V_0 in the methylated region of length a, and another constant value V_1 in the hydroxylated region of length b. The lengths of the regions are proportional to the number of rows of each group (e.g. in Fig. S20, $a/b = 3$). We furthermore assume that $exp(-(V_0 - V_1)/k_B T) = K_0/K_1$, where K_0 is the partition coefficient in the methylated region and K_1 is the partition coefficient in the hydroxylated region. Then, the diffusivity relationship simplifies to:

$$\frac{D}{D_0} = \frac{\gamma(\gamma + (1-\gamma)\varphi)^2(\gamma + (1-\gamma)/\varphi) + (1-\gamma)(1-\gamma+\gamma/\varphi)^2(1-\gamma+\gamma\varphi)}{[\gamma(\gamma + (1-\gamma)\varphi) + (1-\gamma)(1-\gamma+\gamma/\varphi)]^3}$$

Where $\gamma = a/(a + b)$ and $\varphi = K_0/K_1$.

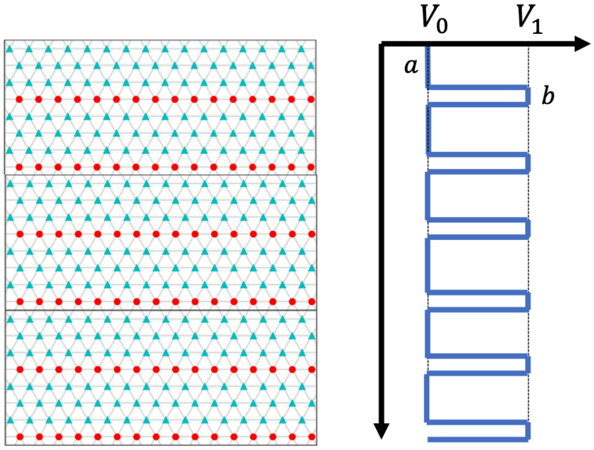

Figure S20: $V(x)$ takes on two distinct values in the methylated and hydroxylated regions.

Finally, to empirically account for the reduction in diffusivity with increasing fraction of hydroxyl groups, we model D_0 as an exponential: $D_0 = Aexp(-B\, f_{OH}) + C$ where f_{OH} is the fraction of hydroxyl groups and the constants $A = 0.1803$, $B = 1.3380$, and $C = 0.0618$ are fit from the computed diffusivities as $f_{OH} = 0$, $f_{OH} = 1$, and the average of diffusivities of the single-patch and dispersed patterns at $f_{OH} = 0.5$.

S5E. Other solutes

Parameters for solutes besides boric acid and arsenous acid are from the GAFF2 forcefield[6] with AM1-BCC charges.[7] For isopropanol and phenol charges are scaled according to the method described in Ref. [[8]], as was performed in Ref. [[9]]. For arsenous acid, non-bonded LJ parameters are from the GAFF2 forcefield, while non-bonded LJ parameters and bonded parameters are from the DREIDING forcefield,[10] with unscaled 1-4 interactions and the harmonic angle potential functional form. We compute partial charges for arsenous acid by running a geometry optimization

and then a single-point electrostatic potential (ESP) calculation in Gaussian 16[11] using the B3LYP functional with the 6-311++G(d,p) basis set. We set the spin multiplicity to 1 for this calculation. We then use AmberTools16[6] to perform a restrained electrostatic potential fitting to fit partial charges for each arsenous acid atom.

Solute	Hydrogen bonds with nanopore hydroxyl groups	$\Delta G_{solv}^{CH3} - \Delta G_{solv}^{OH}$ [kBT]	Flux difference in ringed pore minus flux difference in fully methylated pore $[10^{-6}/ps/Å^2]$
Benzene	0	-3.22	2.64
Phenol	1.15	-3.39	2.35
Isopropanol	1.49	-2.65	0.54
Boric acid	1.27	-0.86	0.011
Ammonia	0.76	-0.73	-0.21
Arsenous acid	1.53	N/A	-0.24

Table S6: For each solute simulated here, the average number of hydrogen bonds formed with the nanopore hydroxyl groups in the fully hydroxylated pore, the difference in affinity for methylated and hydroxylated self-assembled monolayer (SAM) surfaces as measured in Ref. [9], and the enhancement in solute rejection with the ringed pore. The average number of hydrogen bonds with hydroxyl groups in the fully hydroxylated pore is computed from an equilibrium simulation in an infinite pore with the given solute (same workflow as described in the main text for boric acid) and is computed over snapshots for which the solute is within 5Å of the pore wall. Hydrogen bonds are determined via a geometric criteria: the distance between the donor and acceptor oxygen atoms must be less than 3.5Å and the angle formed by the acceptor oxygen – donor oxygen – donor hydrogen must be less than 30°. The enhancement in solute rejection with the ringed pore is computed from subtracting the flux difference (water flux minus solute flux) in the methylated pore from the flux difference in the pore with a single ring of hydroxyl groups (bottom-right pore depicted in Fig. 4). The chosen solutes show a large range of hydrogen-bonding and surface affinity behavior. The difference between their affinities for the methylated and hydroxylated SAMs is correlated with the enhancement in rejection by the ringed pores, as is consistent with the proposed diffusive hopping mechanism.

S5F. Partition coefficients

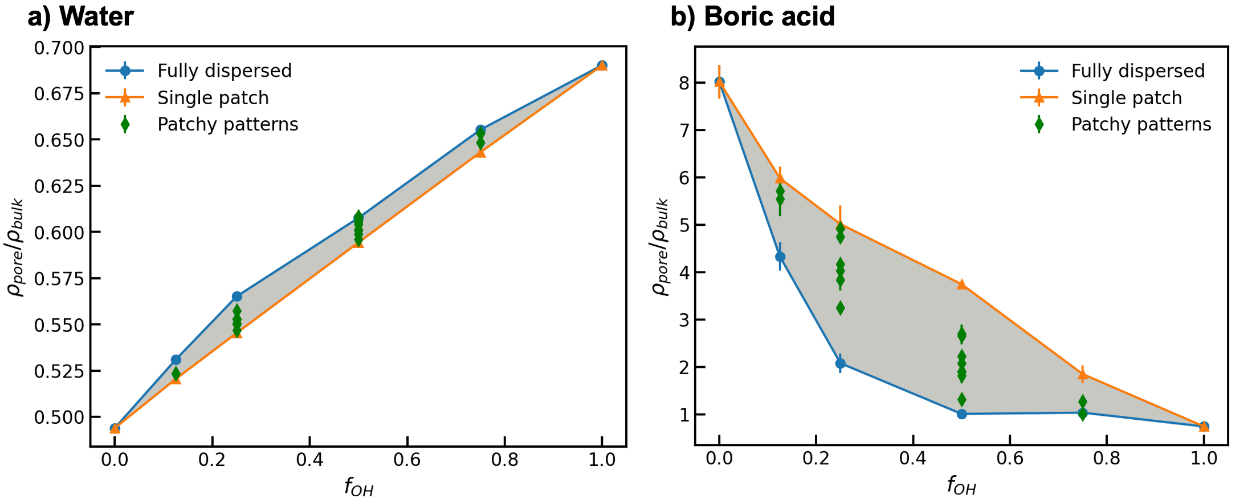

Figure S21: Partition coefficients for a) water and b) boric acid for all rationally designed patterns. Uncertainties are the standard error of the mean from three independent simulations (for the water partition coefficient, they are smaller than the marker size).

S6. Permeability-selectivity trade-off

As described in the Methods section of the main text, water permeability is computed from:

$$P = \frac{K_w F_w V_{pore} \rho_{bulk}}{dP/dz}$$

where K_w is the partition coefficient of water, F_w is the in-pore flux of water, V_{pore} is the pore volume, ρ_{bulk} is the bulk water density, and dP/dz is the pressure gradient (the relationship between the pressure gradient and the applied acceleration is given in section S1H).

Selectivity is computed from:

$$S = \frac{K_w F_w}{K_b F_b} \frac{1}{1.4 \times 10^{-6}}$$

where K_b is the partition coefficient of boric acid, F_b is the in-pore flux of boric acid, and 1.4×10^{-6} is the approximate mole fraction of boric acid in seawater from Ref. [[12]].

In Fig. S22, the Pareto front (shaded gray) shows the set of all optimal combinations of permeability and selectivity from 20 new randomly generated pores (without pattern entropy minimization, i.e. distinct from those used in the genetic algorithm optimization). Here, the Pareto front, an approach to quantify improvement in multi-objective optimization,[13,14] defines pore patterns that lie on the extremes of higher permeability and selectivity. The front crosses from the top-left corner (high selectivity and low permeability) to the bottom right (low selectivity and high permeability), showing that, for randomly generated pores, improvements in selectivity are at the expense of permeability. We compute the permeability and selectivity for the optimal patterns along the convex hull of the genetic algorithm optimization trajectories (Fig. 2, red lines) and determine new Pareto fronts, adding each of the optimal patterns to the original set of 20 randomly generated patterns. For the optimization runs, the best surfaces in terms of flux difference also expand the Pareto front, indicating that the genetic algorithm optimization of a single objective function discovers surfaces that are more optimal in a related, multi-dimensional space, i.e. with more optimal permeability-selectivity behavior. While not directly related, the log of the selectivity contains a term that is the difference of the log of the in-pore fluxes, along with a term related to the difference in partition coefficients and a constant term: $\log S = (\log F_w - \log F_b) + (\log K_w - \log K_b) - \log(1.4 \times 10^{-6})$. Thus, larger flux differences will lead to improved selectivity, as long as the resulting difference in partition coefficients does not offset it.

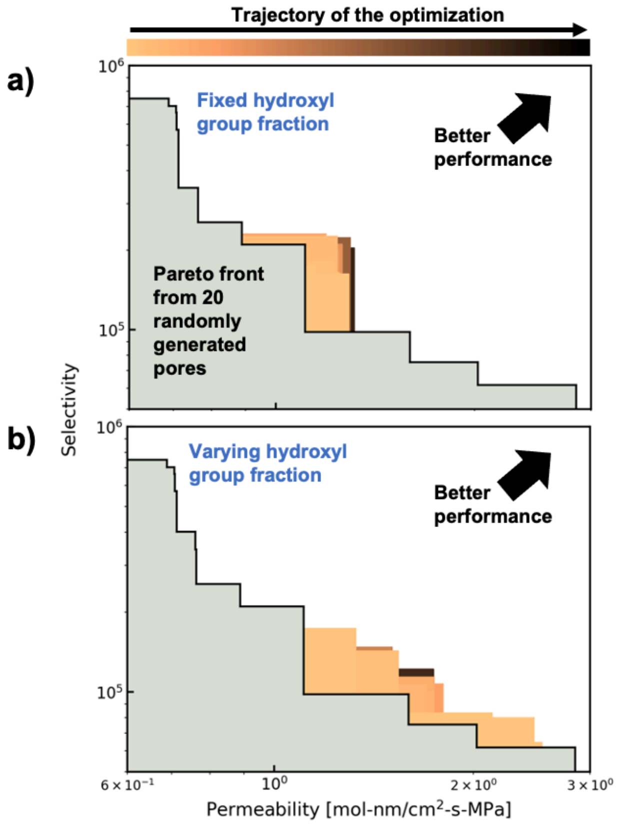

Figure S22: For both the genetic algorithm runs with (a) hydroxyl group fraction fixed at 0.25 and (b) varying hydroxyl group fraction, the optimal patterns along the convex hull extend the Pareto front (borders of tan rectangles) beyond that created from randomly generated pores (black line with gray shading). The darker shades correspond to patterns discovered later in the optimization.

References

(1) Kaminski, G. A.; Friesner, R. A.; Tirado-Rives, J.; Jorgensen, W. L. Evaluation and Reparametrization of the OPLS-AA Force Field for Proteins via Comparison with Accurate Quantum Chemical Calculations on Peptides. *J. Phys. Chem. B* **2001**, *105* (28), 6474–6487. https://doi.org/10.1021/jp003919d.

(2) Risplendi, F.; Raffone, F.; Lin, L.-C.; Grossman, J. C.; Cicero, G. Fundamental Insights on Hydration Environment of Boric Acid and Its Role in Separation from Saline Water. *J. Phys. Chem. C* **2020**, *124* (2), 1438–1445. https://doi.org/10.1021/acs.jpcc.9b10065.

(3) Abraham, M. J.; Murtola, T.; Schulz, R.; Páll, S.; Smith, J. C.; Hess, B.; Lindahl, E. GROMACS: High Performance Molecular Simulations through Multi-Level Parallelism from Laptops to Supercomputers. *SoftwareX* **2015**, *1–2*, 19–25. https://doi.org/10.1016/j.softx.2015.06.001.

(4) Monroe, J. I.; Shell, M. S. Computational Discovery of Chemically Patterned Surfaces That Effect Unique Hydration Water Dynamics. *Proc. Natl. Acad. Sci.* **2018**, *115* (32), 8093–8098. https://doi.org/10.1073/pnas.1807208115.

(5) Reimann, P.; Van den Broeck, C.; Linke, H.; Hänggi, P.; Rubi, J. M.; Pérez-Madrid, A. Giant Acceleration of Free Diffusion by Use of Tilted Periodic Potentials. *Phys. Rev. Lett.* **2001**, *87* (1), 010602. https://doi.org/10.1103/PhysRevLett.87.010602.

(6) AMBER 2016, 2016.

(7) Bayly, C. I.; Cieplak, P.; Cornell, W.; Kollman, P. A. A Well-Behaved Electrostatic Potential Based Method Using Charge Restraints for Deriving Atomic Charges: The RESP Model. *J. Phys. Chem.* **1993**, *97* (40), 10269–10280. https://doi.org/10.1021/j100142a004.

(8) Fennell, C. J.; Wymer, K. L.; Mobley, D. L. A Fixed-Charge Model for Alcohol Polarization in the Condensed Phase, and Its Role in Small Molecule Hydration. *J. Phys. Chem. B* **2014**, *118* (24), 6438–6446. https://doi.org/10.1021/jp411529h.

(9) Monroe, J. I.; Jiao, S.; Davis, R. J.; Robinson Brown, D.; Katz, L. E.; Shell, M. S. Affinity of Small-Molecule Solutes to Hydrophobic, Hydrophilic, and Chemically Patterned Interfaces in Aqueous Solution. *Proc. Natl. Acad. Sci.* **2021**, *118* (1), e2020205118. https://doi.org/10.1073/pnas.2020205118.

(10) Mayo, S. L.; Olafson, B. D.; Goddard, W. A. DREIDING: A Generic Force Field for Molecular Simulations. *J. Phys. Chem.* **1990**, *94* (26), 8897–8909. https://doi.org/10.1021/j100389a010.

(11) Gaussian 16, Revision C.01, 2016.

(12) Hilal, N.; Kim, G. J.; Somerfield, C. Boron Removal from Saline Water: A Comprehensive Review. *Desalination* **2011**, *273* (1), 23–35. https://doi.org/10.1016/j.desal.2010.05.012.

(13) Jablonka, K. M.; Jothiappan, G. M.; Wang, S.; Smit, B.; Yoo, B. Bias Free Multiobjective Active Learning for Materials Design and Discovery. *Nat. Commun.* **2021**, *12* (1), 2312. https://doi.org/10.1038/s41467-021-22437-0.

(14) Janet, J. P.; Ramesh, S.; Duan, C.; Kulik, H. J. Accurate Multiobjective Design in a Space of Millions of Transition Metal Complexes with Neural-Network-Driven Efficient Global Optimization. *ACS Cent. Sci.* **2020**, *6* (4), 513–524. https://doi.org/10.1021/acscentsci.0c00026.
